# The hexapeptide functionalized gold nanoparticles protect against sepsis-associated encephalopathy by forming specific protein corona and regulating macrophage activation

**DOI:** 10.1016/j.mtbio.2025.101704

**Published:** 2025-03-27

**Authors:** Zichen Song, Hongguang Chen, Wenfei Xu, Xiaoye Zong, Xiaoyu Wang, Yuting Ji, Jiameng Gong, Mimi Pang, Shan-Yu Fung, Hong Yang, Yonghao Yu

**Affiliations:** a Department of Anesthesia, Tianjin Institute of Anesthesiology, Tianjin Medical University General Hospital, NO. 154 Anshan Road, Tianjin 300052, China; b Department of Pharmacology and Tianjin Key Laboratory of Inflammation Biology, The Province and Ministry Co-Sponsored Collaborative Innovation Center for Medical Epigenetics, School of Basic Medical Sciences, Tianjin Medical University, No. 22 Qixiangtai Road, Heping District, Tianjin 300070, China; c Department of Immunology and Key Laboratory of Immune Microenvironment and Disease (Ministry of Education), School of Basic Medical Sciences, Tianjin Medical University, No. 22 Qixiangtai Road, Heping District, Tianjin 300070, China

**Keywords:** Sepsis-associated encephalopathy, Bioactive nanoparticles, Protein corona, Monocyte/macrophage, Inflammation

## Abstract

Sepsis-induced systemic inflammatory responses can often lead to brain dysfunction with impaired cognitive function and mobility, known as sepsis-associated encephalopathy (SAE). Currently, there are no effective pharmacological therapeutics to treat SAE. Herein, we demonstrated the hexapeptide functionalized gold nanoparticles P12 that reduced SAE in septic mice with a dual mechanism to down-regulate systemic inflammation. We found that intraperitoneally administered P12 could target macrophages and regulate their inflammatory responses to decrease systemic inflammation and improve mice's cognitive function and mobility with SAE. Depleting peritoneal macrophages diminished the neuroprotective effects of P12 in SAE mice, suggesting macrophages as the effector cells for the neuroprotection by P12. In addition, the proteomic analysis revealed that P12 was capable of sequestering specific circulating inflammatory proteins in the blood of septic mice by forming a protein corona, contributing to the suppression of systemic inflammation. We also found that the local administration of P12 directly to the brain parenchyma effectively inhibited microglia activation and neuroinflammation in mice with SAE. This study provides an insightful understanding of the function and mechanisms of action of P12 in regulating sepsis-associated systemic inflammation and presents a new drug-free nanotherapeutic approach to treat SAE.

## Highlights

• A novel anti-inflammatory and neuroprotective nanodevice P12 is developed.
• P12 improves the cognitive function and mobility of septic mice with SAE.
• P12 targets macrophages and reduces their inflammatory responses in septic mice.
• P12 sequesters inflammatory mediators in circulation by forming a protein corona.
• The local brain injection of P12 reduces neuroinflammation in SAE mice.

## Graphical abstract

**Figure:**
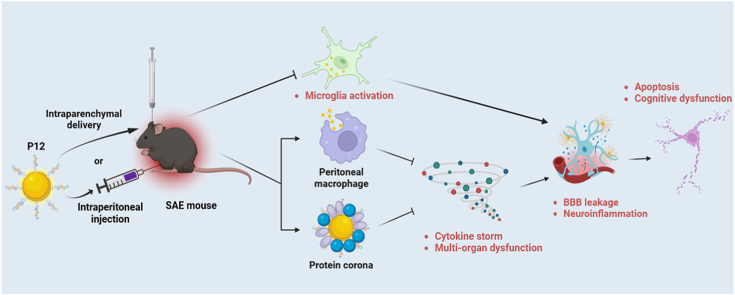

## 1 Introduction

Sepsis, defined as life-threatening organ dysfunction caused by dysregulated host responses to infection, represents one of the main threats to the survival and prognosis of patients in intensive care units [1]. About 30–70 % of septic patients can have the manifestation of brain dysfunction as sepsis-associated encephalopathy (SAE), which is often associated with poor prognosis of patients and can lead to a high economic burden for the health system [2]. Even though patients have recovered from sepsis, their cognitive function, mobility, and quality of life are often impaired for months to years after discharge [2]. However, there is no treatment for this complication; only symptomatic treatment is available clinically [3]. Therefore, effective life-saving and life quality-improving strategies for SAE treatments are essential in critical care medicine to ameliorate the poor prognosis of septic patients.

The pathophysiology of SAE involves a complex interplay of several mechanisms, including brain microvascular injury, neuroinflammation, oxidative stress injury, and neurotransmitter and brain metabolism disorders. Among these potential mechanisms, neuroinflammation plays a critical role in the pathogenesis of SAE [3], which not only causes dysfunction and massive apoptosis of brain cells (e.g., microglia, neurons, and endothelial cells) [4] but induces septic complications with worse outcomes [5]. In addition, SAE is primarily attributed to the systemic inflammatory responses of sepsis rather than the direct infection of the central nervous system (CNS). Thus, attenuating the systemic inflammatory responses associated with sepsis and the subsequent neuroinflammation is a promising strategy to improve long-term cognitive function [6].

It has been reported that the hyperactivation of mononuclear phagocytes, including monocytes and macrophages, plays a key role in the pathogenesis of sepsis and SAE [[7], [8], [9], [10], [11]]. These cells produce and secret many pro-inflammatory mediators, such as tumor necrosis factor-α (TNF-α) and interleukin-1 (IL-1), and are the major initial infiltrating immune cells to CNS in sepsis [12]; activation of infiltrating mononuclear phagocytes and resident microglia in the brain is strongly associated with excessive neuroinflammation [13,14]. The activation of mononuclear phagocytes in sepsis is primarily driven by recognizing microbial products through Toll-like receptors (TLRs) (e.g., endotoxin by TLR4) [15,16]. Specifically, the ligation of TLR4 with its prototypical ligand lipopolysaccharide (LPS) triggers the downstream nuclear factor-kappa B (NF-κB) activation and induces the production of the pro-inflammatory cytokines TNF-α, IL-6, and IL-1β in peritoneal macrophages, which exacerbates inflammation in sepsis [[17], [18], [19]]. Therefore, targeting the mononuclear phagocytes and inhibiting their TLR activation may be an effective approach to control systemic inflammatory responses and neuroinflammation, which ultimately improves cognition in patients with SAE [12,14].

We previously developed a novel class of drug-free, peptide-functionalized bioactive nano-hybrids (P12) that can potently inhibit TLR4 signaling pathway in macrophages [20]. They are made of gold nanoparticles (GNPs)-coated with hexapeptides with the ability to modulate the endosomal acidification process to inhibit TLR4 signaling [21]. Furthermore, they are capable of inducing anti-inflammatory M2 macrophage polarization to promote the resolution of inflammation [22]. Based on these promising features, we hypothesized that P12 can regulate the systemic and brain inflammation in sepsis to alleviate SAE, serving as a new nanotherapeutics for SAE.

To test our hypothesis, we systematically evaluated the anti-inflammatory effects of P12 in two experimental sepsis mouse models and investigated the protective effects of P12 on autonomous locomotion, spatial learning and memory, and cognitive functions of septic mice. From the pathological scores, cytokine analysis, mouse survival rate, and behavioral tests, it was found that P12 significantly protected the septic mice against SAE through down-regulating systemic inflammation. In addition, the stereotaxic injection of P12 into the brains of septic mice confirmed their local neuroprotective effects. Mechanistically, P12 primarily targeted peritoneal macrophages and inhibited their activation and inflammatory reactions to suppress systemic and neuroinflammation in SAE. More importantly, we discovered that P12 specifically adsorbed proinflammatory mediators in the circulation to form a protein corona, contributing to the reduction of systemic inflammation. This study identified a novel strategy for utilizing bioactive nanodevices to reduce the systemic inflammatory responses for treating SAE.

## 2 Materials and methods

### 2.1 Synthesis and characterization of peptide-GNP hybrids

The peptide sequence CLPFFD was screened based on our previous studies [20,21]. The peptide can stabilize GNPs in physiological conditions and form the peptide-GNP hybrids P12. The GNPs and P12 were synthesized based on the method in our earlier work [20]. Briefly, the peptide (CanPeptide Inc., Montreal, Canada) solution (1 mM) was mixed with the synthesized GNP solution (∼11 nM) at a volume ratio of 1:10. The mixture was incubated overnight and sterilized by filtration through a syringe filter (Millipore, Billerica, MA, USA). Unbound peptides in the mixture were removed by centrifugation and washing with the phosphate-buffered saline (PBS) (G4202, Servicebio, Beijing, China) three times. The precipitated hybrids were then resuspended in a culture medium or PBS at a desired concentration for further use. The morphology and size of the bare GNPs and P12 were characterized by a JEOL JEM-2100 transmission electron microscope (Tokyo, Japan). To fabricate SS and TT nanohybrids, the peptide sequences of CLPSSD and CLPTTD were synthesized, and their aqueous solutions were incubated with GNPs following the same protocol.

### 2.2 Culture of microglia and neuronal cells in vitro

BV-2 microglia and HT22 neuronal cells were purchased from Procell (Wuhan, China). The cells were cultured in DMEM (Procell, Wuhan, China) supplemented with 10 % fetal bovine serum (Kangyuan, Beijing, China) and antibiotics (100 IU/mL penicillin and 100 μg/mL streptomycin, Sigma, St. Louis, MO, USA) in a humidified incubator at 37 °C with 5 % CO_2_. Cells were stimulated with LPS (1 μg/mL) (*Escherichia coli 055:B5*, Servicebio, Beijing, China) and treated with P12 (5 nM) for 24 h for TEM analysis or 0–2 h for immunoblotting analysis. To evaluate the toxicity of P12 on both cells, cells were treated with P12 at different concentrations (1–25 nM) for 24 h, followed by an MTS assay (Keygenbiotech, Jiangsu, China). HT22 cells were incubated with the conditional media of BV-2 cells under 24-h stimulation of LPS (1 μg/mL), and treated with P12 (5 nM) for 24 h before the MTS assay to assess the cell viability.

### 2.3 Western blotting analysis

After treatments, cultured cells were washed twice with prechilled PBS and lysed with prechilled RIPA buffer on ice for 20 min. The cell lysates were centrifuged (14,000 rpm, 4 °C, 10 min), and the supernatants were collected to determine the total protein concentration using a BCA protein kit (Beyotime, Nanjing, China). After adjusting the protein concentration, proteins were separated by 10 % SDS-PAGE and transferred to a PVDF membrane (Millipore, Billerica, MA, USA). The membrane was blotted with primary antibodies against phosphorylated p65 (cat#3033), IκBα (cat#9242), and β-actin (cat#8457) (all from Cell Signaling Technology, Danvers, MA, USA) at 4 °C overnight, and then incubated with HRP-labeled secondary antibodies (Promega, Madison, USA) at room temperature for 1 h before the development of chemiluminescence (Millipore, Burlington, MA, USA). The protein bands were imaged on a ChemiDoc MP imaging system (Bio-Rad, Hercules, CA, USA). The densitometry of the protein bands was analyzed using the ImageJ software (NIH, Bethesda, USA).

### 2.4 SAE mouse models

C57BL/6J male mice (SPF) at 7–10 weeks old were used for all in vivo experiments. For the behavioral tests, 8–10-week-old mice were used with one week of habituation in the test environment; 7–9-week-old mice were used for all the remaining experiments. The mice were randomly assigned to different experimental groups. Animal care and handling procedures followed the Guide for the Care and Use of Laboratory Animals. The animal protocols were approved by the Institutional Animal Care and Use Committee of Tianjin Medical University General Hospital (IRB2023-DW-113).

Sepsis was induced either by the cecal ligation and puncture (CLP) procedure or through intraperitoneal (i.p.) injection of LPS. CLP was performed according to the following procedures. Mice were anesthetized with 3 % sevoflurane by inhalation for the operation of a median abdominal incision. The cecum was identified and ligated with a 4-0 nylon thread at the distal end. The ligated cecum was punctured once with a 21G needle to release a small amount of feces, and it was then placed back into the abdominal cavity. After the operation, mice were given saline (1 mL) subcutaneously and observed until they recovered from anesthesia. Three doses of P12 (500 nM, 100 μL) were given through i.p. injection at 2, 8, and 14 h after CLP. At 24 h after CLP, mice were euthanized, and the blood and organs were collected for further experiments. In another set of experiments, mice were observed over 10 days for the survival analysis. To evaluate the therapeutic effects of SAE, mice were tested for their behaviors to assess their cognitive function and mobility on Days 1, 3, and 7 after CLP.

For the LPS-induced SAE mouse model, mice were challenged with LPS (10 mg/kg) (*Escherichia coli 055:B5*, Servicebio, Beijing, China) through i.p. injection; P12, Cy5-labeled P12, or PBS was administered intraperitoneally at 2, 8, and 14 h after LPS challenge. At 24 h, mice were anesthetized and perfused with saline, and the brains were collected and imaged on an IVIS Spectrum Imaging System (Xenogen, GA, USA). In another set of experiments, mice were tested for their behaviors to evaluate their cognitive function and mobility on Days 1, 3, and 7 after the LPS challenge. On Day 7, mice were examined for vascular permeability in the brain and lungs.

To deplete the peritoneal macrophages, clodronate (Clo) liposomes (5 mg/mL, 200 μL, GlpBio, Montclair, USA) were injected intraperitoneally 24 h before the lethal LPS (40 mg/kg) challenge through i.p. injection. After P12 treatment (500 nM, 100 μL) at 2, 8, and 14 h after the LPS challenge, mice were observed over 6 days for survival rate. In another setting, a lower dose of LPS (10 mg/kg) was given intraperitoneally to assess the effects of P12 on the behaviors of SAE mice with/without peritoneal macrophage depletion.

### 2.5 Behavioral tests on the SAE mice

The behavioral tests were performed in both CLP- and LPS-induced SAE mouse models on Days 1, 3, and 7 for the open field test (OFT), Y-maze test, and new object recognition test (NORT), respectively. The objects and field were cleaned with 75 % ethanol between each trial to eliminate the olfactory cues. Supermaze video tracking software was used to record the data.

The open field test box is 50 × 50 × 25 cm in dimension and divided into nine (3 × 3) equal squares. It was placed in a quiet, dark environment. The movement distance, walking speed, and resting time of the mice in the box were recorded within 5 min after they were put into the box.

The Y-maze test consisted of three arms (start, novel, and another arm) (40 cm × 4.5 cm × 12 cm) placed at 120° to each other. The mice were subjected to a Y-maze test twice with a 2-h interval to evaluate spatial recognition memory. The first trial (training) allowed the mouse to explore 2 arms (start and other) freely for 10 min. Two hours after the first trial, mice were free to explore all three arms of the maze for 5 min. The number of entries, distance moved, and duration in the novel arm were analyzed.

For NORT, each mouse was placed in a square open field (50 × 50 × 25 cm) and allowed to explore the open field with two identical objects located in opposite and equidistant positions for 10 min. After a 2-h retention interval, the mouse was allowed to explore the open field again for 5 min, where one of the two objects was replaced by a novel object. The discrimination index and the number of exploring the novel object were analyzed. Discrimination index = (time spent on the novel object)/(total time spent on both objects) × 100 %.

### 2.6 The analysis of the protein corona on the bare GNPs and P12

Twenty-four hours after the LPS challenge (or PBS as the control), mouse blood was collected from the heart by cardiac puncture and placed in microcentrifuge tubes with 100 μL of sodium citrate. The blood samples were left at room temperature for 1 h and centrifuged at 4000 rpm for 5 min to collect the supernatant as the blood plasma. The bare GNPs and P12 suspensions (25 nM) were added dropwise to the plasma to obtain the ratio of the plasma volume of 30 μL per nanoparticle surface area of 1 cm^2^; the mixtures were incubated at 37 °C for 4 h.

The mixtures were centrifuged and washed with PBS three times (12,000 rpm, 10 min, 4 °C) to remove the unbound proteins, and the pellets were treated with SDT sample buffer (4 % SDS, 100 mM Tris/HCl, 1 mM DTT, pH 7.6) to elute proteins adsorbed on the nanoparticles. The samples were under shaking for 3 min and sonicated in a water bath for 40 min, followed by centrifugation at 14,000 rpm for 30 min. The supernatants were collected for the mass spectrometry analysis of the protein compositions in the protein corona.

### 2.7 LC-MS/MS and data analysis

The LC-MS/MS analysis of the extracted proteins from the protein corona was performed by Shanghai APTBIO Co. (Shanghai, China). The data were processed by Spectronaut software (Spectronaut™ 14.4.200727.47784) using the same library construction database. The filtering parameter Q value cutoff was set to 0.01 (equivalent to FDR <1 %). The differentially enriched proteins (FC > 1.5 for up-regulation and FC < 0.67 for down-regulation, *P* < 0.05) were grouped and categorized using the Hierarchical Cluster algorithm and presented in the Heatmap (top 30 proteins). The Principal Component Analysis (PCA) was performed using R language (pca and ggplot2 R package). The Venn diagram was plotted through the website (http://bioinformatics.psb.ugent.be/webtools/Venn/) (FC > 1.5 for up-regulation and FC < 0.67 for down-regulation, *P* < 0.05). Comparison groups with differentially enriched proteins were presented by volcano plots and proteins with FC > 10 were individually labeled. The KAAS (KEGG Automatic Annotation Server) software was utilized to perform KEGG pathway analysis. Fisher's exact test was used to compare the distribution of KEGG pathways in the target protein set with the overall protein set and for the enrichment of KEGG pathway annotations in the target protein set.

### 2.8 Cytokine analysis

The levels of the cytokines TNF-α, IL-6, and IL-1β in the serum and hippocampus from the SAE mice were determined by ELISA kits (eBioscience, San Diego, CA) following the manufacturer's instruction. The concentration of cytokines in the tissues was normalized to the total proteins.

BV-2 cells were seeded in 24-well plates (4 × 10^5^ cells/well) and rested for 24 h. The cells were treated with P12 (5 nM) and LPS (1 μg/ml) for 4 h. The supernatant of the culture medium in each group was collected and stored at −20 °C before the analysis. The cytokine IL-6 (eBioscience, San Diego, CA) was measured by the ELISA kit following the manufacturer's instructions.

### 2.9 TUNEL staining

The brain sections were processed and stained with anti-NeuN antibodies (cat#ab177487, Abcam, Cambridge, MA, USA) to label neurons. The TdT-mediated dUTP Nick-End Labeling (TUNEL) staining was conducted on the mouse brain tissue sections to assess the cell apoptosis by BrightRed Apoptosis Detection Kit (Vazyme, Nanjing, China). The cell nuclei were stained with DAPI (Roche, Basel, Switzerland). The stained amygdaloid cells were imaged on a fluorescence microscope (OLYMPUS, BX53, Tokyo, Japan).

### 2.10 Isolation of the peritoneal macrophages

The efficiency of peritoneal macrophage depletion by Clo liposomes was evaluated by counting the number of macrophages in the peritoneal lavage fluid collected 24 h after the LPS challenge. The sterile PBS (5 mL) was injected into the abdominal cavity, and the peritoneal lavage fluid was collected after massaging the abdomen for 30 s; this procedure was repeated three times. The collected fluid was centrifuged (300 g) for 5 min, and the cell pellets were resuspended in PBS for total cell counting using a hematocrit analyzer after red blood cell lysis with 1.5 % acetic acid. Aliquots of the cell suspensions (∼3 × 10^4^ cells) were processed with Cytospin (StatSpin, USA) on a glass slide, which was stained with Liu stain (Baso Diagnostics, Zhuhai, China) for macrophage counting under a microscope (OLYMPUS, BX53, Tokyo, Japan). More than 200 cells were counted for each slide.

The cells collected from the peritoneal lavage fluid were cultured in a 12-well plate (2.5 × 10^5^ cells/well) with the complete RPMI-1640 medium containing 10 % fetal bovine serum (FBS), 1 mM sodium pyruvate and 2 mM L-glutamine (Gibco, USA) at 37 °C for 6 h. After discarding the non-adherent cells, the adherent peritoneal macrophages were treated with Cy5-labeled P12 at 37 °C for 4 h. The cells were washed with PBS twice, fixed, and stained with cytosolic DiO for the cell membranes and DAPI for the nuclei; the stained cells were then imaged on a confocal microscope (OLYMPUS, FV1200, Tokyo, Japan) to observe the uptake of P12.

### 2.11 Assessment of the permeability of BBB and the lung in SAE mice

The permeability of the blood-brain barrier (BBB) and the lung was assessed using the Evans blue (EB) extravasation method. In the LPS-induced SAE mouse model, one week after the LPS challenge, EB dye (2 % w/v) was intravenously injected into mice at a dose of 4 mL/kg. Two hours after EB administration, mice were perfused extensively with saline and then euthanized to collect the brain and lungs. The cerebrum of the brain was divided into hemispheres, each of which was homogenized and sonicated in 1 mL of 50 % trichloroacetic acid, followed by centrifugation at 12,100 g for 20 min. Right lungs were homogenized in formamide (1 mL/100 mg lung) and incubated for 24 h at 37 °C. Homogenates were centrifuged (12,000 g) for 20 min, and the absorbance (at 620 nm) of the supernatant (200 μL) was measured in a 96-well plate on a microplate reader (Thermo Scientific, Waltham, USA).

### 2.12 Hematoxylin and eosin staining

The mouse organs were harvested, fixed with 10 % formalin, embedded in paraffin, sectioned and stained with hematoxylin and eosin (H&E), and imaged on a microscope (OLYMPUS, BX53, Tokyo, Japan) for pathological analysis. The lung and liver histological scores were analyzed by two independent investigators in a blinded manner. Lung injury was assessed in six randomly selected areas of each section based on the five different features: (i) neutrophils in the alveolar space, (ii) neutrophils in the interstitial space, (iii) the presence of hyaline membranes, (iv) protein fragments filling the air spaces, and (v) thickened alveolar septa. The liver injury was classified into four grades according to the intensity of necrosis and spread of inflammatory cells: 0, no necrosis or inflammation; 1, mild hepatocellular necrosis with a mild inflammatory response; 2, diffuse hepatocellular necrosis and intrafollicular necrotic bridges with the inflammatory response; 3, destruction of lobules and diffuse hepatic necrosis with the diffuse interfollicular inflammatory response.

### 2.13 Local treatment of P12 by stereotactic injection to the brain of SAE mice

To evaluate the direct effects of P12 on neuroinflammation in the brain of SAE mice, P12 was given through stereotactic injection 30 min after LPS challenge through i.p. injection (10 mg/kg). Mice were anesthetized with isoflurane and placed on a stereotactic headstock, and P12 (2 μL, 500 nM) or an equal volume of saline was injected into the cortex and hippocampus. Twenty-four hours after the LPS challenge, the mouse brain was harvested for TEM imaging to observe the P12 uptake in the brain and for immunofluorescence staining to examine the activation of microglia.

For the immunofluorescence staining, mice were deeply anesthetized and transcardially perfused with saline and 10 % formalin. After that, the mouse brain was harvested, fixed, and embedded in paraffin. The brain sections were processed and stained with anti-Iba-1 antibodies (cat#ab283319, Abcam, Cambridge, MA, USA) for microglia activation. The cell nuclei were stained with DAPI. The fluorescence signals were acquired on a fluorescence microscope (OLYMPUS, BX53, Tokyo, Japan).

### 2.14 Transmission electron microscopy (TEM)

The LPS-induced SAE mouse model was used to investigate the uptake of P12 in the brain of SAE mice with three treatments of P12 by i.p. injection at 2, 8, and 14 h after the LPS challenge. To study the diffusion of P12 in the brain of SAE mice, P12 was given by stereotactic injection 30 min after the LPS challenge. Brain tissues of approximately 1 cubic millimeter were collected 24 h after the LPS challenge. The uptake of P12 in neurons and microglia was assessed in vitro on HT22 and BV-2 cells, respectively. The cells were treated with P12 (5 nM) for 24 h, and then collected and centrifuged at 1000 rpm for 5 min. The cell pellets or the brain tissues were fixed in 2.5 % glutaraldehyde for 24 h. The samples were then incubated in osmium tetroxide for 2 h to stabilize the phospholipid membranes of the cells and organelles. The samples were then dehydrated, washed with PBS, and embedded in resin. Samples were cut into ultrathin sections (70 nm), which were stained with 4 % uranyl acetate and 0.04 % lead citrate on 200 mesh copper grids. Images were captured on a TEM (Hitachi H-7800, Japan).

### 2.15 Hydrodynamic sizes and zeta potential of the nanohybrids

After synthesis, the hydrodynamic diameter and the polydispersity index (PDI) of P12 were measured by the dynamic light scattering (DLS) technique on a Zetasizer (Malvern Instruments, Worcestershire, UK). Different nanohybrids (P12, TT, and SS) were incubated with the proteins C1qα or MMP7 (Cloud-Clone Corp., Wuhan, China) overnight. The changes in hydrodynamic sizes and zeta potentials before and after incubation were analyzed by a Zetasizer (Malvern Instruments, Worcestershire, UK).

### 2.16 Statistical analysis

All data were presented as the mean ± standard error of the mean (SEM). Statistical analysis was performed using one-way ANOVA or Student's t-test by GraphPad Prism (9.0.0) whenever applicable unless otherwise specified. *P* < 0.05 was considered statistically significant.

## 3 Results

### 3.1 The neuroprotective activity of the peptide-functionalized nano-hybrid P12 in sepsis mice

**Fig. 1:**
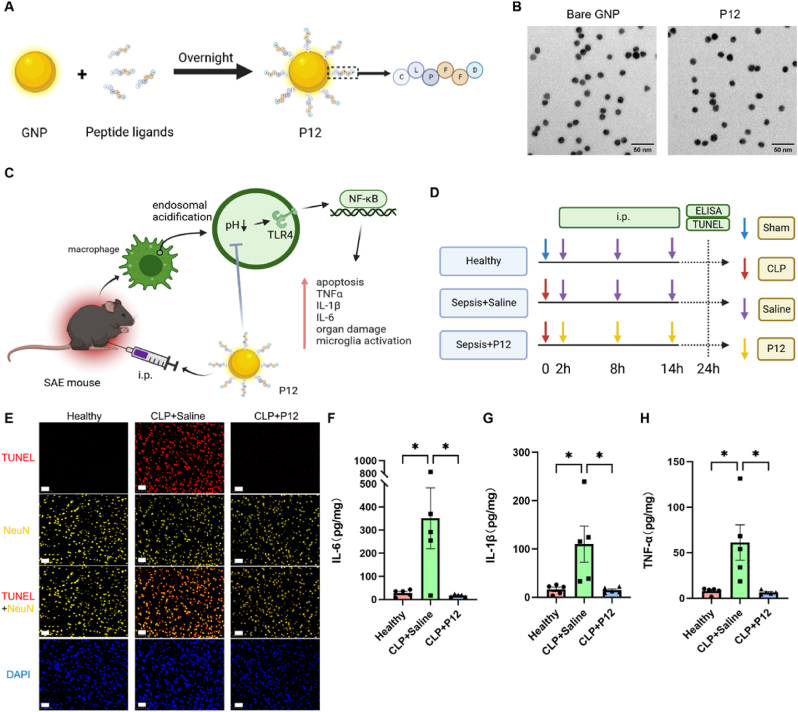
**Synthesis of the peptide-GNP hybrid P12 and its anti-inflammatory effects in the brain in the CLP-induced SAE mouse model.** (A) A schematic diagram showing the synthesis of the peptide-coated GNP P12; the decorating peptide has a sequence of CLPFFD. (B) TEM images of the unmodified GNPs (the bare GNP) and the peptide-coated GNPs (P12); scale bar = 50 nm. (C) A schematic diagram of the proposed working mechanism of P12 upon intraperitoneal (i.p.) injection for treating SAE. (D) A scheme showing the CLP-induced SAE mouse model; P12 (500 nM, 100 μL) was given through i.p. injection at 2, 8, and 14 h after CLP. (E) The fluorescence microscopic images of NeuN-stained (yellow) and TUNEL-stained (red) amygdaloid cells collected from the mice 24 h after CLP. The nuclei were stained with DAPI (blue); scale bar = 50 μm; N = 3–4/group. (F–H) The levels of the cytokines IL-6 (F), IL-1β (G), and TNF-α (H) in the hippocampus were measured by ELISA at 24 h after CLP; N = 5/group. ∗*P* < 0.05; illustrations in (A), (C), and (D) were created with the help of BioRender.com.

In our previous study, a novel cargo-free anti-inflammatory nano-hybrid (P12) was developed, which is made of a GNP core with a diameter of 13 nm coated with hexapeptides on the surface (Fig. 1A) [20]. The TEM images showed that P12 was monodispersed with a spherical structure similar to the bare GNPs (Fig. 1B). The hydrodynamic size of P12 was found to be 18.6 ± 0.1 nm (PDI: 0.09 ± 0.01) by DLS analysis (Fig. S1). Since P12 was found to inhibit TLR4 signaling and downstream inflammatory responses in macrophages [21], we hypothesized that it may regulate the inflammatory macrophages to suppress systemic inflammation in sepsis to exert cerebroprotective effects in SAE mice (Fig. 1C).

To test this overarching hypothesis, we applied the cecal ligation and puncture (CLP) procedure to induce SAE in mice for evaluating the neuroprotective effects of P12 in the brain; mice were treated with P12 by intraperitoneal injections at 2, 8, and 14 h post-operation (Fig. 1D). At the end of the model, we performed TUNEL staining on the brain tissue sections of mice and found that the increased cell apoptosis in the amygdala of SAE mice was significantly attenuated by P12 treatment (Fig. 1E and Fig. S2A). In addition, the elevated production of the proinflammatory cytokines IL-6, IL-1β, and TNF-α in the hippocampus tissues of SAE mice was significantly reduced by P12 (Fig. 1F–H). In the surrounding tissue of the hippocampus region, the increased TNF-α level in the CLP mice was decreased by P12, but the levels of IL-6 and IL-1β were not affected by CLP or P12 (Figs. S2B–D). It is worth noting that the hippocampus is more severely injured during SAE when compared with other regions in the brain by clinical observation [[23], [24], [25]]. Thus, the observed anti-inflammatory activity of P12 in the hippocampus tissue of SAE mice is clinically important. Taken together, these results suggested that P12 exhibited neuroprotective activity in SAE mice by reducing amygdala apoptosis and significantly decreasing proinflammatory cytokine production in the brain.

**Fig. 2:**
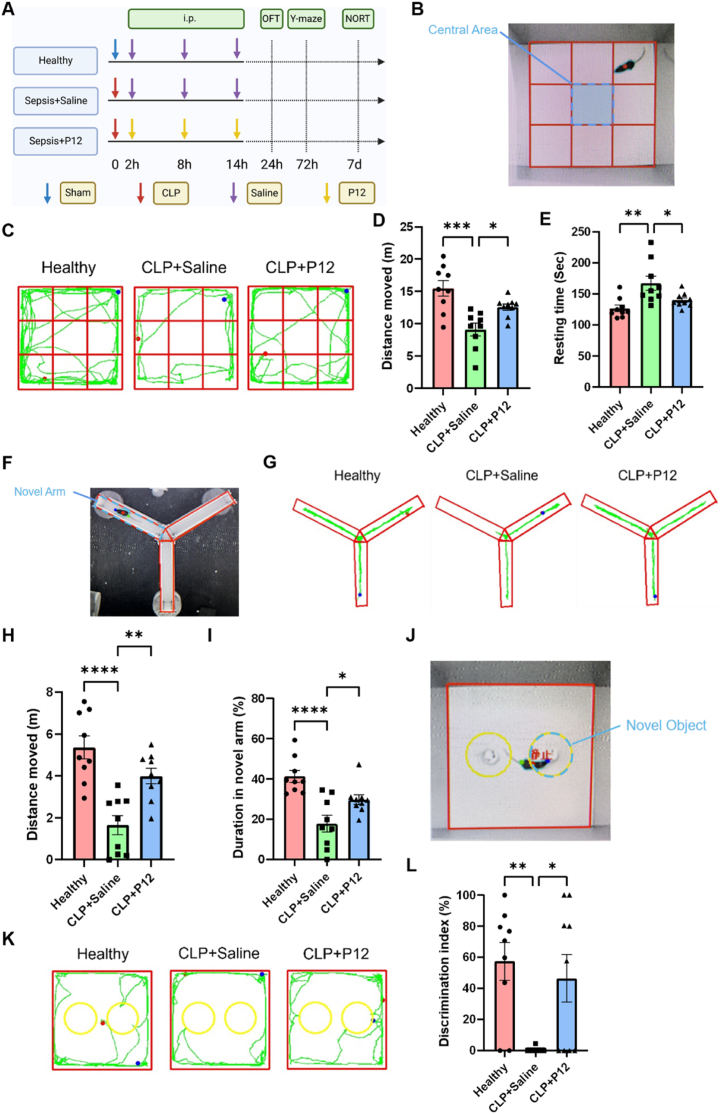
**The effects of P12 on the mouse behavior in the CLP-induced SAE mouse model.** (A) The scheme showing the CLP-induced SAE mouse model; P12 was administered through the i.p. injection at 2, 8, and 14 h after CLP; at 24 h, 72 h, and 7 day after CLP, mice were assessed for the open field test (OFT), Y-maze test, and novel object recognition test (NORT), respectively; the illustration was created with the help of BioRender.com. (B) The representative picture showed the top view of the open field test. (C) Representative records of the walking paths of mice (green line) from different groups in the OFT; the starting and end points of the mouse movement were shown as blue and red dots, respectively. (D, E) The quantitative analysis of the walking distance (D) and resting time (E) of mice in the OFT. (F) The representative photo showed the top view of the Y-maze. (G) Representative records of the walking paths of mice from different groups in the Y-maze test. (H, I) The quantitative analysis of the walking distance (H) and duration (I) of mice in the novel arm of the Y-maze test. (J) The representative photo showed the top view of the NORT. (K) The representative records of the walking paths of mice from different groups in the NORT. (L) The quantitative analysis of the discrimination index of mice from each group in the NORT. N = 9/group; ∗*P* < 0.05, ∗∗*P* < 0.01, ∗∗∗*P* < 0.001, ∗∗∗∗*P* < 0.0001.

Next, we wondered whether such neuroprotective effects of P12 had significant impacts on the behaviors of SAE mice. For this purpose, mice were subjected to the open field test (OFT), the Y-maze test, and the novel object recognition test (NORT) on Days 1, 3, and 7 after CLP induction, respectively (Fig. 2A). In the OFT analysis, the CLP-induced SAE mice had impaired movements as expected; the P12 treatment significantly increased the distance traveled by SAE mice (Fig. 2B–D), suggesting the capability of P12 in improving the voluntary locomotor ability of SAE mice. In addition, P12 was able to decrease the resting time of SAE mice (Fig. 2E), suggesting that P12 could attenuate the anxiety-depression-like behaviors of SAE mice. In the Y-maze test, it was found that P12 increased the distance and duration of the novel arm exploration of SAE mice (Fig. 2F–I). We further calculated the total distance traveled by each group of mice in the Y-maze and found that the CLP mice had a significant decrease in distance traveled, which was significantly improved by the administration of P12 (Fig. S3A). To exclude the effect of decreased locomotor ability for cognitive tests, we calculated the distance ratio in the novel arm of mice in each group and found that the P12 treatment group had a significantly lower ratio than that of the control group, suggesting that P12 could significantly improve the recognition memory and spatial reference memory in SAE mice (Fig. S3B). For NORT, the total distance traveled by each group of mice is similar (Fig. S3C). Thus, the NORT revealed that the P12 treatment effectively increased the discrimination index of SAE mice in recognizing novel objects against the background of the same motor ability in all groups of mice, indicating that the loss in learning and cognitive function of SAE mice could be reverted by P12 (Fig. 2J–L). Taken together, these results demonstrated that P12 was capable of alleviating motor and memory impairments and cognitive dysfunction in the SAE mice.

**Fig. 3:**
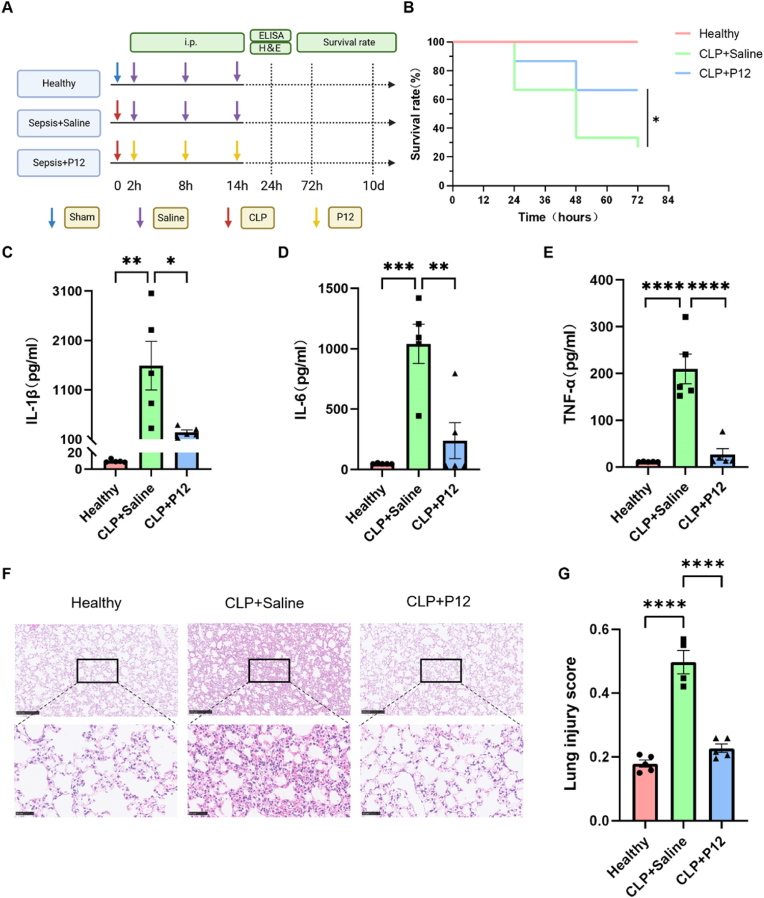
**The effects of P12 on mouse survival, the systemic inflammatory responses, and acute lung injury in mice with CLP-induced sepsis.** (A) The scheme showed the CLP-induced sepsis mouse model; the systemic inflammatory responses were evaluated at 24 h after CLP, whereas the mouse survival rate was monitored for up to 10 days after CLP; the illustration was created with the help of BioRender.com. (B) The survival rates of the septic mice with/without the P12 treatment at 72 h after CLP; N = 15/group; the log-rank (Mantel-Cox) test was used for the statistical analysis. (C–E) The levels of the cytokines IL-6 (C), IL-1β (D), and TNF-α (E) in the mouse serum were measured by ELISA at 24 h after CLP; N = 5/group. (F) The representative histological images of the mouse lung tissue sections with H&E staining at 24 h post CLP; scale bars = 250 μm for top panels and 50 μm for bottom panels. (G) The lung injury score was analyzed from (F) in each group; N = 4–5/group. ∗*P* < 0.05, ∗∗*P* < 0.01, ∗∗∗*P* < 0.001, ∗∗∗∗*P* < 0.0001.

As systemic inflammation is the key causing multi-organ dysfunction and SAE in sepsis, we then assessed the effects of P12 on the systemic inflammatory responses and multi-organ damages in a long-term (10 days) CLP-induced sepsis mouse model (Fig. 3A). It was found that P12 significantly increased the survival rate of the septic mice over 72 h (Fig. 3B); however, the 10-day survival rate was not affected by P12 (Fig. S4), suggesting that P12 exhibited a potent protective effect at the early phase (up to 72 h) of CLP-induced sepsis. Indeed, we found that the elevated proinflammatory cytokines IL-1β (Fig. 3C), IL-6 (Fig. 3D), and TNF-α (Fig. 3E) in the serum of the septic mice were significantly down-regulated by P12 at 24 h. We also observed the reduction in acute lung inflammation and injury by P12 from the histological images and scoring of lung tissues of the septic mice (Fig. 3F and G). Moreover, the CLP-induced acidophilic degeneration and protein cast in the kidneys were decreased upon the P12 treatment (Fig. S5). Notably, the lungs and kidneys emerged as the initial sites of inflammatory injury at 24 h post-CLP, while other organs (e.g., heart, mesenteric lymph nodes, brain, and liver) demonstrated no significant histopathological alterations (Fig. S5).

### 3.2 The anti-inflammatory and neuroprotective activities of P12 in the LPS-induced SAE mice

**Fig. 4:**
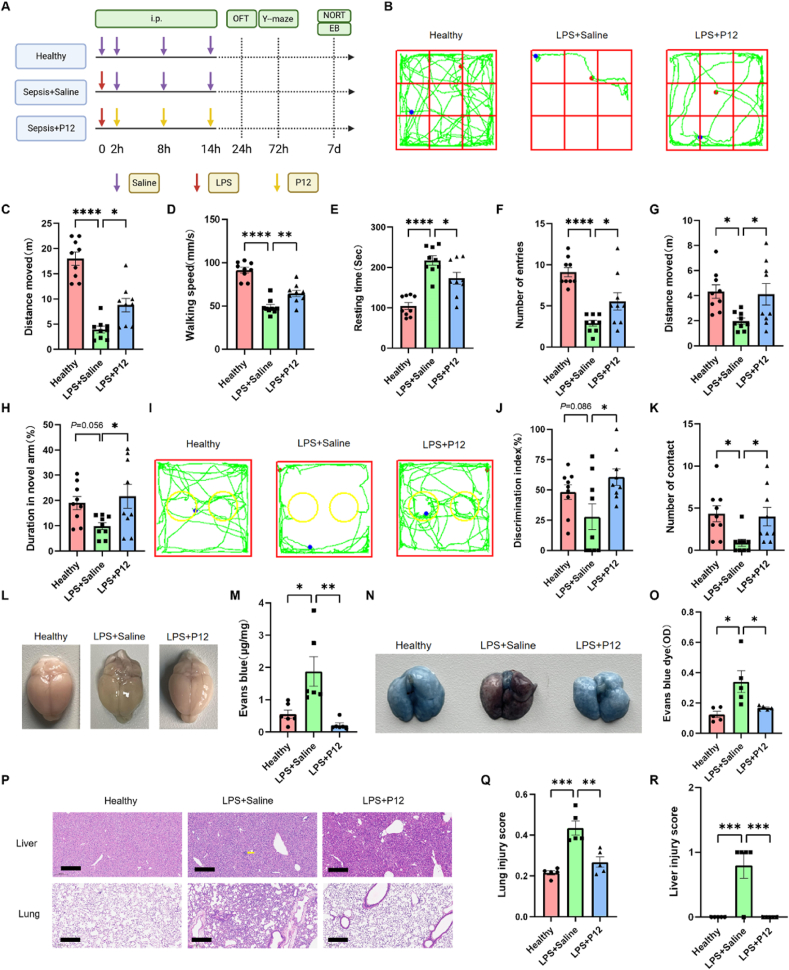
**Effects of P12 on the mouse behavior and systemic inflammation in the LPS-induced SAE mouse model.** (A) The scheme showing the LPS-induced SAE mouse model; LPS (10 mg/kg) was given by i.p. injection and P12 was administered through the same route at 2, 8, and 14 h after LPS challenge; at 24 h, 72 h, and 7 day after LPS challenge, mice were assessed for the OFT, Y-maze test, and NORT, respectively; the illustration was created with the help of BioRender.com. (B) The representative records of the walking paths of mice (green line) from different groups in the OFT; the starting and end points of the mouse movement were shown in blue and red dots, respectively. (C–E) The quantitative analysis of the walking distance (C), mean speed (D), and resting time (E) of mice in the OFT. (F–H) The quantitative analysis of the number of entries (F), walking distance (G), and duration (H) of mice in the novel arm of the Y-maze test. (I) The representative records of the walking paths of mice from different groups in the NORT. (J, K) The quantitative analysis of the discrimination index (J) and the number of exploring the novel object (K) of mice from each group in the NORT; N = 9/group for all behavioral tests. (L, M) The BBB permeability characterized by the Evans blue (EB) diffusion test on Day 7 after the LPS challenge; the representative images of the brain shown in (L) and the quantified EB staining (EB/total proteins, μg/mg) in (M); N = 6/group. (N) The representative images of the EB-stained lungs of mice. (O) The quantification of EB in the lungs in (N). (P) The representative histological images showing the characteristics of the liver (top) and lung (bottom) injuries in mice; yellow arrows indicated the punctate necrosis; scale bar = 200 μm. (Q, R) The injury scores of the lung (Q) and the liver (R) were analyzed from (P) for each group; N = 5/group. ∗*P* < 0.05, ∗∗*P* < 0.01, ∗∗∗*P* < 0.001 ∗∗∗∗*P* < 0.0001.

In addition to the CLP model, we employed another SAE mouse model induced by i.p. injection of LPS to evaluate the effects of P12 on SAE under gram-negative bacteria infection (Fig. 4A); the P12 treatment was given through the same route at 2, 8 and 14 h after LPS challenge. The behavioral analysis of LPS-induced SAE mice revealed that the P12 treatment enhanced both cognitive function and mobility in the SAE mice as demonstrated by the performance improvements in the OFT (Fig. 4B–E), the Y-maze test (Fig. 4F–H; Figs. S6A and B), and the NORT (Fig. 4I–K and Fig. S6C). These findings were consistent with those from the CLP model (Fig. 2).

The integrity of the blood-brain barrier (BBB) in the SAE mice on Day 7 was examined by the Evans blue (EB) dye diffusion. It was seen that the brain of the SAE mice displayed the blue color but that of the P12-treated ones did not (Fig. 4L). The quantification of EB absorption in the brain homogenates showed that P12 treatment significantly reduced the leakage of EB dye in the brain (Fig. 4M). These results indicated that P12 was effective in preventing BBB disruption to reduce neuroinflammation and neuronal damage in SAE mice. Furthermore, the P12 treatment was able to reduce the vasculature leakage in the lung as probed by the EB dye in LPS-induced septic mice (Fig. 4N and O) as well.

We also examined the effects of P12 on the multi-organ damage in LPS-induced sepsis. It was observed that the LPS challenge caused serious lung inflammation and injuries, and various degrees of necrosis in the liver and mesenteric lymph nodes, which were reduced by the P12 treatment (Fig. 4P–R; Fig. S7). Of note, the lungs and liver exhibited the earliest histopathological evidence of inflammatory injuries in this model, while other organs (e.g., heart, brain, and kidneys) did not show any significant change (Fig. S7). These results demonstrated that P12 was capable of attenuating systemic inflammation and organ damage in mice with sepsis.

### 3.3 P12 acted on peritoneal macrophages to exert protective effects on brain function in SAE mice

**Fig. 5:**
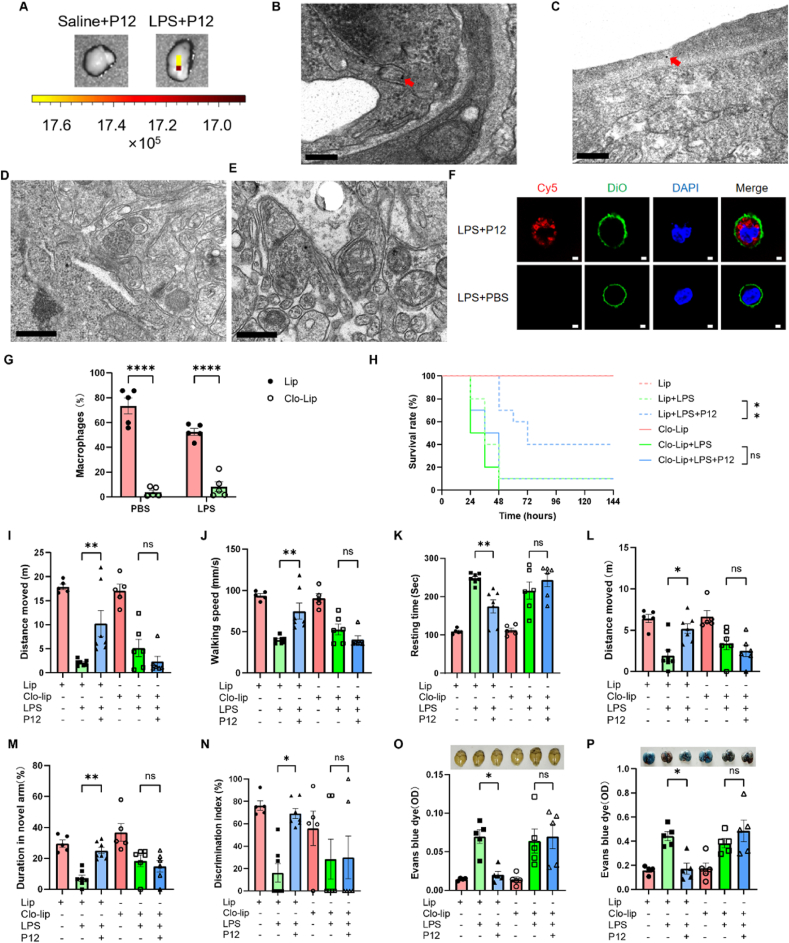
**Intraperitoneally administered P12 targeted peritoneal macrophages to reduce LPS-induced SAE in mice.** (A) Ex vivo fluorescence images of the brains from the healthy and SAE mice treated with Cy5-labeled P12 (500 nM, 100 μL) for 24 h through i.p. injection. (B–E) The representative TEM images showing the presence of P12 (by red arrows) in brain endothelial cells (B, C) in the hippocampal (B) and cortical (C) regions, but not in the brain parenchyma (D, E) in the hippocampus (D) and cortex (E) of SAE mice 24 h after i.p. injection of P12; scale bar = 200 nm for (B, C) and 500 nm for (D, E); N = 2/group. (F) Confocal microscopic images of peritoneal macrophages incubated with Cy5-labeled P12 (50 nM) at 37 °C for 4 h; DiO (green) was used to label the cell membrane and DAPI (blue) for the nuclei; scale bar = 2 μm; N = 3/group. (G) The analysis of the percentage of peritoneal macrophages in the peritoneal lavage fluid upon the depletion by clodronate-liposomes (200 μL per mouse) for 48 h through i.p. injection 24 h before the LPS challenge (10 mg/kg); blank liposomes were used as the vehicle control; N = 5/group. (H) The effects of P12 treatment on the survival rate of septic mice challenged with the lethal dose of LPS (40 mg/kg) with/without peritoneal macrophage depletion; N = 10/group; the log-rank (Mantel-Cox) test was used for the statistical analysis. (I–K) The effects of P12 on the walking distance (I), mean speed (J), and resting time (K) in the OFT of mice with LPS-induced sepsis with/without peritoneal macrophage depletion. (L, M) The effects of P12 on the walking distance (L) and duration (M) in the Y-maze test of the mice with LPS-induced sepsis with/without peritoneal macrophage depletion. (N) The effects of P12 on the discrimination index in the NORT of the mice with LPS-induced sepsis with/without peritoneal macrophage depletion; N = 5–7/group for all the tests. (O, P) The effects of P12 on the vascular leakage in the brain (O) and lungs (P) of mice with LPS-induced sepsis by the EB diffusion assay with/without peritoneal macrophage depletion; the representative images were shown at the top; N = 4–5/group. LPS = 10 mg/kg except for the mouse survival test; ns: not significant; ∗*P* < 0.05, ∗∗*P* < 0.01, ∗∗∗∗*P* < 0.0001.

Since P12 showed potent effects on reducing neuroinflammation and improving brain function in the SAE mice, we wondered whether P12 could get into the brain by passing through the BBB after i.p. administration. To answer this question, we investigated the localization of Cy5-labeled P12 after i.p. injection in mice with LPS-induced SAE. Under the in vivo imaging system, a strong fluorescence signal of Cy5 was observed in the brains of Cy5-P12 treated SAE mice when compared with the PBS-treated SAE mice (Fig. 5A), indicating the accumulation of P12 in the brain during SAE. However, further TEM imaging on the brain tissues revealed that P12 was only present in the endothelial cells (indicated by the red arrows) of the hippocampal and cortical regions of the brains (Fig. 5B–E), suggesting that the intraperitoneally administered P12 could not cross the BBB to directly act on the inflammatory neurons or microglia in the brain.

As P12 did not have direct action in the brain to alleviate SAE, we speculated that it probably acted on the peritoneal macrophages to control the systemic inflammation for the prevention of cerebral damage during sepsis. To test our hypothesis, we first examined the cellular uptake of Cy5-P12 in the peritoneal macrophages in vitro. It was seen the presence of the red fluorescence of Cy5-P12 inside the peritoneal macrophages when compared with the PBS-treated control upon LPS stimulation (Fig. 5F), demonstrates the targeting ability of P12 to the peritoneal macrophages.

Next, we depleted the peritoneal macrophages by i.p. injection of clodronate liposomes (Clo-lip) 24 h before the LPS challenge to investigate whether the peritoneal macrophages were the effector cells through which P12 exhibited anti-inflammatory and neuroprotective activities. The peritoneal lavage fluids (PLF) were collected 24 h after the LPS challenge for the assessment of the macrophage depletion efficiency by differential cell counting (Fig. S8). It was found that the percentage of peritoneal macrophages in the PLF cells was lower than 10 % after Clo-lip treatment (Fig. 5G), indicating successful depletion of macrophages in the peritoneal cavity. When the peritoneal macrophages were depleted, the protective effects of P12 on the increased survival rate of septic mice were diminished (Fig. 5H). In addition, the depletion of peritoneal macrophages also abrogated the therapeutic effects of P12 on the neurobehavioral performance of SAE mice in the OFT (Fig. 5I–K; Fig. S9A), the Y-maze test (Fig. 5L and M; Fig. S9B), and the NORT (Fig. 5N; Fig. S9C). Moreover, the P12 treatment did not reduce the vasculature leakage in the brain (Fig. 5O) and in the lungs (Fig. 5P) with the absence of the peritoneal macrophages. These results suggested that the intraperitoneal administered P12 primarily acted on the peritoneal macrophages to exert the anti-inflammatory and neuroprotective effects in SAE mice.

### 3.4 The capability of P12 in scavenging specific inflammatory mediators in the blood of SAE mice

**Fig. 6:**
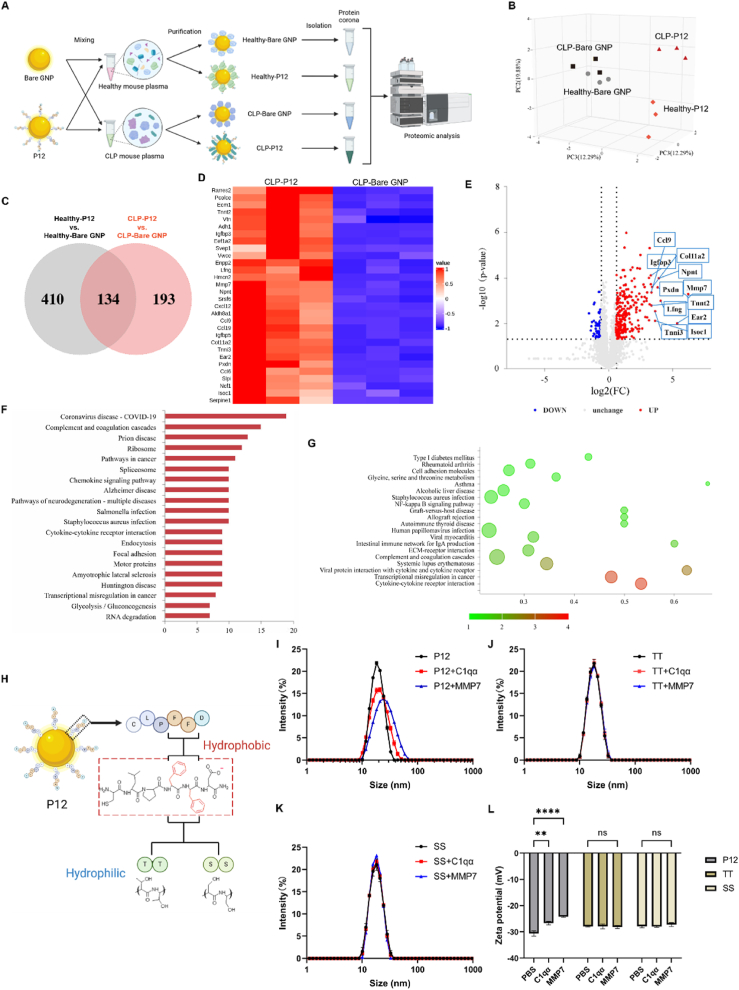
**The analysis of the protein corona on P12 and the bare GNPs mixed with the blood plasma collected from healthy or septic mice.** (A) A scheme showing the experimental design to analyze the protein corona on P12 and the bare GNPs when mixed with the blood plasma of the healthy mice or the mice with CLP-induced sepsis; the illustration was created with the help of BioRender.com. (B) The principal component analysis (PCA) demonstratinging the sample clustering among different groups: CLP-bare GNP, CLP-P12, healthy-bare GNP, and healthy-P12. (C) The Venn diagram showing the differentially enriched plasma proteins by P12 vs. the bare GNP. (D) The heatmap showing the differentially enriched proteins (Top30) of CLP-P12 vs. CLP-bare GNP. (E) The volcano plot displaying the differentially enriched proteins of CLP-P12 vs. CLP-bare GNP (>1.5 FC for up-regulated proteins and ＜0.67 FC for down-regulated proteins; *P* value < 0.05); the proteins with FC > 10 were labeled. (F) The KEGG pathway analysis of differentially enriched proteins showing the Top20 up-regulated pathways by CLP-P12 vs. CLP-bare GNP. (G) The bubble plot of the enriched pathways of the differentially enriched proteins in CLP-P12 vs. Healthy-P12 from the KEGG pathway enrichment analysis. (H) A scheme showing the hexapeptides conjugated on the GNP surface; the two amino acid residues FF of the decorating peptides on P12 were replaced with the hydrophilic residues threonine (T) or serine (S) to become the inactive nanohybrid TT or SS, respectively; the illustration was created with the help of BioRender.com. (I–K) The hydrodynamic sizes of P12 (I), TT (J), and SS (K) before and after mixing with the protein C1qα or MMP7 by DLS analysis. (L) The changes in zeta potential values of P12, TT, and SS after overnight incubation with C1qα or MMP7. N = 3 independent experiments; ns: not significant; ∗∗*P* < 0.01, ∗∗∗∗*P* < 0.0001.

In addition to regulating peritoneal macrophages to reduce systemic inflammation, we also suspected that P12 may adsorb certain plasma proteins to form the protein corona, by which many circulating inflammatory mediators are neutralized, contributing to the reduction of systemic inflammation in septic mice. To test this hypothesis, we performed the proteomic analysis on the protein corona formed on the bare GNP and P12 that were incubated with the blood plasma collected from the healthy mice or the CLP-induced septic mice for 4 h (Fig. 6A). The principal component analysis (PCA) showed that the samples of the bare GNP were clustered closely together regardless of the blood plasma from healthy or septic mice; however, the samples of P12 were away from the bare GNP groups, and distinctly separated into two clusters depending on the blood plasma from healthy or septic mice (Fig. 6B). This suggested that when compared with the bare GNP, P12 could interact with specific plasma proteins, and such selection was affected by the inflammatory condition in mice. From the Venn diagram analysis, it was found that 327 proteins were differentially enriched in the CLP-P12 group in comparison with the CLP-bare GNP group, while 544 proteins were differentially enriched in the healthy condition of P12 vs. the bare GNP (Fig. 6C). The top 30 differentially enriched proteins of CLP-P12 vs. CLP-bare GNP were presented in the heatmap (Fig. 6D), in which several proteins were involved in the inflammatory responses such as the matrix metalloproteinase 7 (MMP7), C-C motif chemokine 9 (CCL9), CCL6 and CXCL12. This was also clearly seen in the volcano plot (Fig. 6E), where P12 had a stronger affinity to grab CCL9, MMP7, and insulin-like growth factor-binding protein (IGFBP) than the bare GNP under CLP condition. It should be noted that macrophage inflammatory protein 1γ (MIP-1γ, or CCL9) is a chemokine secreted by macrophages and myeloid cells to trigger macrophage motility and infiltration during inflammation [26]; MIP-1γ can also function through its cell surface receptor CCR1 to induce MMP production that facilitates the endothelial barrier disruption [27,28]. Thus, scavenging MMP7 and CCL9 by P12 may serve as a novel mechanism of action in reducing the systemic inflammatory responses and protecting the endothelial barriers in the brain and lungs as previously observed in the SAE mice.

We further analyzed the populations of the differentially enriched proteins by P12 based on their subcellular locations using CELLO [29]. When comparing P12 with the bare GNP under the CLP condition or comparing the CLP with the healthy condition for P12, the enriched proteins were primarily annotated as the extracellular (∼26–27 %), cytoplasmic (∼25–26 %) and nuclear (∼27–28 %) proteins (Fig. S10). The gene ontology (GO) enrichment for the cellular component (CC) classification also showed that the top 5 enriched CC classifications were in the extracellular regions (Fig. S11). This indicated that P12 indeed had a high affinity to sequester the extracellular proteins that participate in systemic inflammatory responses. Furthermore, the GO enrichment analyses for the biological process (BP) classification revealed that the enriched proteins specific to P12 were mainly classified into the processes related to the defense responses as well as the humoral immune responses (Fig. S12).

The pathway analysis based on the Kyoto Encyclopedia of Genes and Genomes (KEGG) databases showed that the specifically enriched plasma proteins of the septic mice by P12 (vs. the bare GNP) were strongly associated with the complement and coagulation cascades (e.g., complement 1qα, C1qα), chemokine signaling pathways, bacterial infections, cytokine-cytokine receptor interaction and endocytosis (Fig. 6F, Fig. S13). In addition to the above pathways, we also found that the NF-κB signaling pathway was identified from the enriched proteins by P12 under the CLP condition (vs. the healthy condition) (Fig. 6G), supporting our previously observed inhibitory activity of P12 on TLR-mediated NF-κB activation [30]. Altogether, these findings indicated that P12 was capable of regulating the systemic inflammatory responses during sepsis by scavenging the plasma proteins participating in complement activation and cytokine/chemokine signal transduction.

To further confirm the specificity of P12 in interacting with the identified MMP7 and C1qα, we employed two other peptide-GNP hybrids TT and SS that were fabricated with the mutated peptides CLPTTD and CLPSSD (to replace the FF motif) as the inactive control nanoparticles, respectively (Fig. 6H). These nanohybrids were incubated with C1qα and MMP7 overnight, and their hydrodynamic size and zeta potential were measured before and after the incubation. It was found that the hydrodynamic size of P12 increased about 1 nm and 5 nm after incubation with C1qα and MMP7, respectively (Fig. 6I). In contrast, the hydrodynamic sizes of the inactive control TT and SS remained unchanged upon mixing with the proteins (Fig. 6J and K). Furthermore, the zeta potential of P12 was significantly elevated upon the addition of C1qα and MMP7, confirming the adsorption of the proteins on P12 (Fig. 6L). However, the zeta potential of TT and SS remained unchanged after mixing with C1qα or MMP7, suggesting the lack of adsorption of these proteins on their surface (Fig. 6L). These observations demonstrated that the identified composition of the protein corona was specific and unique to P12, and such specificity and selection on the protein corona formation were highly dependent on the hydrophobic FF region of the decorating peptides.

### 3.5 Local treatment of P12 in the brain reduced neuroinflammation in SAE mice

**Fig. 7:**
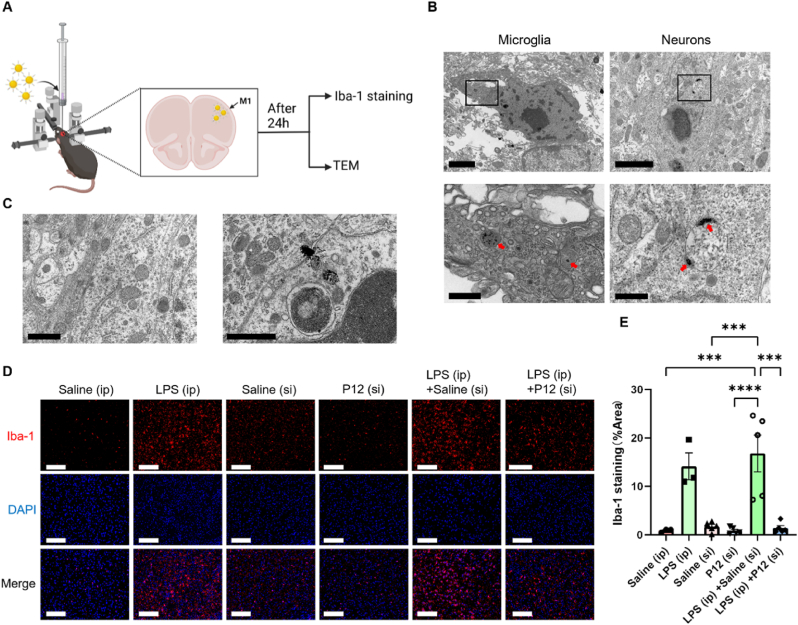
**The effects of P12 on the microglia activation in the brain after stereotactic injection to the SAE mice.** (A) A schematic diagram showing the experimental process of the stereotactic injection of P12 (500 nM, 2 μL) directly to the primary motor cortex (M1) in the brain of the SAE mice upon LPS (10 mg/kg) challenge by i.p. injection; brain tissues were collected and processed for Iba-1 staining and TEM imaging 24 h after LPS challenge; the illustration was created with the help of BioRender.com. (B, C) The representative TEM images showing the presence of P12 (red arrows) in the microglia and neurons in the brains of SAE mice (B), but absence in the healthy mice (C); scale bar = 2 μm (B, top), scale bar = 500 nm (B, bottom), scale bar = 1 μm (C); N = 2/group. (D) The immunofluorescence microscopic images of cortical cells in the brain sections with Iba-1 staining (red) at 24 h post-LPS challenge; the nuclei were stained with DAPI (blue); scale bar = 100 μm. (E) The quantitative analysis of the percentage of Iba-1 positive area in the field from the fluorescence images in (D) by ImageJ software; N = 3–5/group; ∗∗∗*P* < 0.001, ∗∗∗∗*P* < 0.0001.

While the intraperitoneal administration of P12 exerted systemic anti-inflammatory and neuroprotective effects in the SAE mice, we also wondered whether P12 could act directly on the brain to reduce neuroinflammation when locally administered. To answer this question, P12 was given directly into the primary motor cortex of the brain in LPS-induced SAE mice via stereotactic injection (s.i.) to monitor the uptake of P12 by microglia and neurons in the brain and the effects on the microglial activation (Fig. 7A). Twenty-four hours after the injection, the TEM images clearly showed that P12 (indicated by the red arrows) was present in both microglia and neurons in the brain of the SAE mice (Fig. 7B), but not in the cells of the brain of healthy mice (Fig. 7C). This may be because the inflammatory condition facilitated the accumulation of P12 in the brain parenchyma or the activated microglia/neutrons had a higher capacity of phagocytosis/endocytosis of P12 during SAE. The microglia activation in the primary motor cortex and hippocampus was also evaluated by immunofluorescence staining of Iba-1; it was found that the Iba-1 expression was significantly elevated upon LPS challenge, which was effectively decreased by P12 (Fig. 7D and E; Fig. S14). These results demonstrated that the direct injection of P12 into the brain facilitated the uptake of P12 by microglia and neurons during SAE, and down-regulated the microglia activation.

**Fig. 8:**
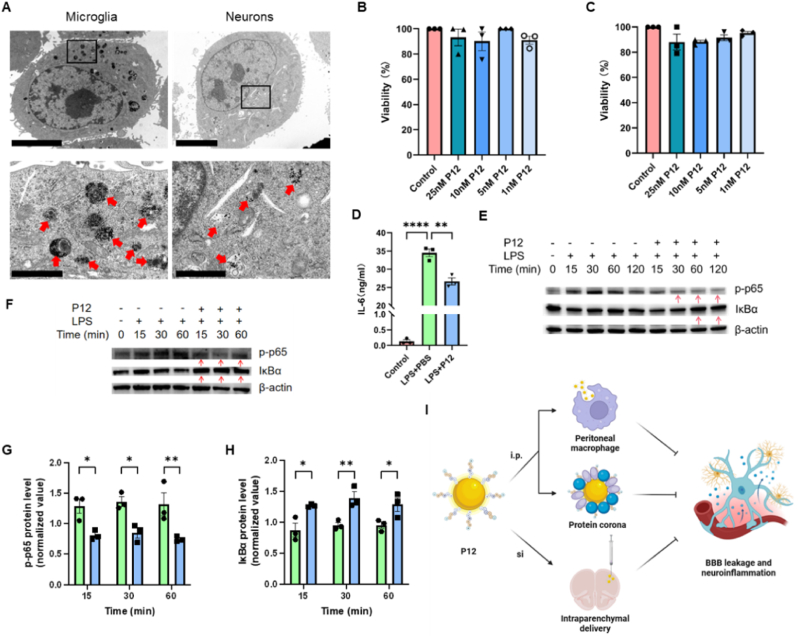
**The effects of P12 on the inflammatory responses in cultured microglia and neurons.** (A) The representative TEM images showing the internalization of P12 (red arrows) in the cultured microglia (BV-2) and neurons (HT22) in vitro; cells were treated with P12 (5 nM) for 24 h; the zoom-in image of the black box was shown at the bottom; scale bar = 5 μm (top) and 1 μm (bottom). (B, C) The effects of P12 at different concentrations on the viability of microglial (BV-2) (B) and neuronal (HT22) (C) cells. (D) The effects of P12 on the IL-6 production of BV-2 cells upon LPS (1 μg/mL) stimulation for 4 h. (E, F) Immunoblots showing the effects of P12 on the phosphorylation of p65 (p-p65) and IκBα degradation for NF-κB activation in BV-2 (E) and HT22 (F) cells upon LPS (1 μg/mL) stimulation for different periods (0–2 h); β-actin as the internal control. (G, H) The densitometry analysis of phosphorylated-p65 (G) and IκBα (H) levels in (F); P12 = 5 nM; N = 3 biological replicates. (I) A scheme showing the proposed working mechanisms of P12 in alleviating SAE in septic mice: i) the intraperitoneally injected P12 targeted peritoneal macrophages and regulated their activation to reduce the systemic inflammation and improve the neurobehavioral performance in septic mice; ii) P12 was able to sequester specific proteins (e.g., CCL9 and MMP) in the blood plasma by forming the protein corona, which facilitated P12 internalization by macrophages and reduction of the systemic inflammation; iii) local injection of P12 directly to the brain down-regulated microglia activation and reduced neuroinflammation; the illustration was created with the help of BioRender.com. ∗*P* < 0.05, ∗∗*P* < 0.01, ∗∗∗∗*P* < 0.0001.

We then examined the anti-inflammatory effects of P12 on the cultured microglial (BV-2) and neuronal (HT22) cells in vitro. Upon the P12 treatment for 24 h, we observed the presence of P12 in both BV-2 and HT22 cells (Fig. 8A). While P12 could be internalized by both cells, P12 did not cause any toxicity to BV-2 (Fig. 8B) and HT22 (Fig. 8C) cells over a wide range of P12 concentrations. It was found that P12 was able to reduce the inflammatory responses in microglial cells by down-regulating IL-6 production (Fig. 8D) and the NF-κB activation (p65 phosphorylation and IκBα degradation) upon LPS stimulation (1 μg/mL) at different time points (Fig. 8E). We also assessed the direct effects of P12 on the neuronal cells and found that P12 was able to inhibit p65 phosphorylation and IκBα degradation under LPS stimulation at different time points as well (Fig. 8F–H). Collectively, these findings suggested that P12 was capable of directly regulating inflammatory responses in both microglia and neurons.

In this study (Fig. 8I), we demonstrated that the intraperitoneal administration of P12 can regulate the activation of peritoneal macrophages to exert systemic anti-inflammatory effects in SAE mice. In the meanwhile, by forming a specific protein corona, P12 can scavenge many plasma proteins associated with complement and coagulation activation, chemokines/cytokines signaling, endocytosis, and NF-κB signaling pathway to reduce the systematic inflammatory responses during sepsis. Furthermore, P12 can directly act on microglia and neurons when locally applied to the brain to control neuroinflammation during SAE. All these pieces of evidence demonstrate that P12 could serve as a potent novel nano-therapy to regulate systemic and neuronal inflammation to treat SAE.

## 4 Discussion

Sepsis-associated encephalopathy (SAE) is a common complication in septic patients characterized by brain dysfunction associated with impaired cognitive function and mobility, which are mainly caused by systemic inflammatory responses during sepsis. Currently, there is no effective treatment to prevent SAE. It is known that macrophages play critical roles in the pathogenesis of sepsis by producing large amounts of proinflammatory cytokines. Thus, specific inhibition of macrophage activation and the associated systemic inflammation is expected to be beneficial for treating SAE. In this study, we showed that the intraperitoneal administration of the drug-free nanodevice P12 (with potent TLR inhibitory activity) protected mice from SAE-associated pathological brain damage, reduced the cognitive dysfunction and systemic inflammation, and increased the survival rate of SAE mice. Furthermore, direct application of P12 to the brain of sepsis mice also exhibited local neuroprotective effects. A dual mechanism was discovered for this novel therapeutic activity of P12: i) P12 primarily targeted macrophages and inhibited their activation and inflammatory reactions; ii) P12 specifically adsorbed proinflammatory proteins in the circulation to form the “protein corona” surrounding the nanoparticle surface to scavenge the systemic inflammatory mediators. Such a novel dual action of immunomodulation makes these peptide-coated GNPs potential next-generation anti-inflammatory therapeutics for treating SAE.

### 4.1 Possible mechanisms of action of P12 for reducing systemic inflammation and neuron damage in SAE

In this study, we found that P12 protected mice from SAE through a dual mechanism. First, P12 targeted macrophages and inhibited TLR4 signaling to control dysregulated macrophage activation and systemic inflammation in mice with SAE. We previously found that P12 can specifically suppress the activation of the key transcription factors NF-κB and IRF3 in the TLR4 signaling pathways triggered by the prototypical ligand LPS in macrophages [20,22]. Accordingly, P12 can effectively attenuate the LPS-induced lung inflammation [31]. These facts support our hypothesis that P12 targets macrophages to inhibit their TLR4 signaling and downstream inflammatory reactions, contributing to protection against SAE. Through the behavioral tests, we did find that P12 exhibited potent anti-inflammatory and neuroprotective effects in mice with SAE. By depleting the peritoneal macrophages, the effects of P12 on improving voluntary movement, learning memory, and cognitive function in SAE mice were significantly lost (Fig. 5), suggesting that the peritoneal macrophages were the effector cells by which P12 exerted its anti-inflammatory activity in SAE.

Second, P12 could adsorb specific inflammatory proteins in the blood of septic mice to form a “protein corona”, to reduce certain circulating inflammatory mediators and systemic inflammation (Fig. 6). The concept of the “protein corona” surrounding nanoparticles was readily accepted in 2007 [32,33]. The presence of the protein corona has been shown to improve the therapeutic effectiveness and safety of nanodevices [34]. In addition, it can impact the cellular recognition [[35], [36], [37]], cellular/molecular targeting capacity [38,39], and immunomodulatory ability [[40], [41], [42], [43]] of the nanodevices by altering their surface properties. Our proteomic analysis of the protein corona on P12 revealed substantial adsorption of CCL9 and MMP7 by P12. The binding of CCL9 to its cell surface receptor CCR1 can trigger the migration and infiltration of macrophages during inflammation, and facilitate the disruption of the endothelial barrier by inducing MMP production. Therefore, the formation of the protein corona to sequester specific inflammatory mediators represented a novel mechanism of action by which P12 attenuated the systemic inflammatory responses and protected the endothelial barrier in SAE mice (Fig. 4L–O). Moreover, we also identified many complement proteins and proteins associated with endocytosis pathways (e.g., the EH domain-containing protein 2, EHD2) in the protein corona on P12 (Fig. 6F and G, Figure S13), which could facilitate the targeting capability of P12 to phagocytic immune cells [[44], [45], [46], [47]] with higher uptake through macropinocytosis [48]. Once P12 was engulfed by these cells, the adsorbed circulating inflammatory proteins in the protein corona on P12 could be degraded in lysosomes, contributing to the decrease in systemic inflammation. Such a mechanism of action provided a novel reasonable explanation for the observed systemic anti-inflammatory effects of P12 in SAE.

### 4.2 Importance of reducing systemic inflammation in SAE

Excessive systemic inflammation in sepsis is the primary factor contributing to the onset of SAE [49], which prompts investigations to control systemic inflammation to treat SAE. It has been reported that non-steroidal anti-inflammatory drugs (NSAIDs) can effectively manage SAE by inhibiting cyclooxygenase (COX) to mitigate the inflammatory responses [50,51]; however, the therapeutic efficacy of NSAIDs in treating SAE is currently debatable [52,53]. On the other hand, molecular hydrogen (H_2_) has been found to have neuroprotective effects by attenuating systemic inflammation and preventing neuronal apoptosis by regulating the TLR4/NF-κB pathway and the NRF2 pathway, respectively [[54], [55], [56]]. Unfortunately, the limited bioavailability and efficacy of hydrogen due to its high diffusivity and low solubility further restricted its uses for treating SAE. Although immunoglobulin (Ig) is capable of suppressing the systemic inflammation of SAE patients [[57], [58], [59], [60], [61]], its high treatment cost, unknown mechanism of action, and limited effectiveness bring caution to its clinical uses for treating SAE [[62], [63], [64], [65]]. Nevertheless, these studies indicate that controlling systemic inflammation appears to be an effective therapeutic strategy for SAE.

Mononuclear phagocytes serve as a significant contributor to systemic inflammation and play a crucial role in the pathogenesis of SAE. Multiple studies have demonstrated that the disruption of BBB in septic mice can lead to the infiltration of inflammatory monocytes into the brains and the activation of microglia [12,66]. In addition, it has been found that the peripheral inflammatory monocytes themselves can impair motor learning and learning-related dendritic spine plasticity through TNF-α-mediated mechanisms [67]. Furthermore, the prevention of inflammatory monocyte infiltration decreases the activation of microglia and neuroinflammation, ultimately alleviating cognitive impairment in mice with SAE [12]. Our results demonstrated that P12 targeted peritoneal macrophages and suppressed their inflammatory responses (Fig. 5). P12 was able to sequester inflammatory mediators in the circulation (Fig. 6), which could reduce the migration of inflammatory monocytes to the brain. Such actions together mitigated systemic inflammation and alleviated SAE by preventing BBB disruption, reducing TNF-α levels in the brain, and enhancing cognitive function (Fig. 1, Fig. 2, Fig. 3, Fig. 4).

### 4.3 P12 as new nanomedicine for SAE treatment

It is known that the deterioration in the function of the amygdala, cortex, and hippocampus of the brain plays an essential role in the pathogenesis of SAE. In SAE patients, the size of the amygdala was reduced with the observed neuronal apoptosis in the amygdala autopsies [23,68]. The cortex has been linked to cognitive impairments in SAE patients [49]. Furthermore, neuroimaging studies have shown that the hippocampus and frontal cortex volumes in SAE patients were reduced during hospitalization and 6–24 months after discharge [23,24]. Interestingly, we found that P12 effectively mitigated the pathological alterations in the amygdala, cortex, and hippocampus of the brain in SAE mice: the P12 treatment substantially reduced cytokine levels in the hippocampus and decreased neuronal apoptosis in the amygdala (Fig. 1E–H); furthermore, the microglial activation in the cortex and hippocampus were suppressed with the local treatment of P12, confirming its direct action on reducing neuroinflammation and ameliorating brain dysfunction in SAE mice (Fig. 7D and E; Fig. S14). These results demonstrated the therapeutic effectiveness of P12 on the pathological alterations in the brain of SAE, serving as a new generation of nanomedicine for the treatment of SAE.

Although it has been found that unmodified GNPs could reduce cerebral microvascular inflammation in septic mice [3], P12 is more advantageous than bare GNPs. First, P12 has superior stability to the bare GNPs (forming large aggregates) at physiological conditions due to the peptide modification [69,70]. Second, P12 has a specific protein corona composition different from the unmodified GNPs (Fig. 6B) to regulate the levels of inflammatory proteins in circulation (Fig. 6D and E). Third, P12 can potently down-regulate the levels of the proinflammatory cytokines IL-6 and IL-1β in the brain of septic mice while the bare GNPs cannot. Fourth, P12 inhibits NF-κB activation in microglia and neurons and increases survival rates of septic mice (Fig. 3B; Fig. 8D–H), surpassing the effects of the unmodified GNPs [3]. Fifth, our previous study has shown that P12 has a good biosafety profile without any acute toxicity at both therapeutic and high doses in vivo [71]; in this study, we also found that P12 did not cause any toxicity to neurons and microglia at the experimental concentrations. Collectively, P12 emerges as a safe and effective GNP-based nanotherapeutics for treating SAE.

## 5 Conclusions

SAE is a cerebral dysfunction caused by systemic inflammatory responses in sepsis, which currently lacks effective treatments. In this study, we developed a bioactive nanodevice, P12. We demonstrated that P12 protected septic mice from SAE by a dual mechanism of action to control the systemic inflammation during sepsis: i) P12 targeted peritoneal macrophages and suppressed their inflammatory responses; ii) P12 sequestered many inflammatory proteins in the circulation system through the formation of a specific protein corona. Moreover, upon direct application to the brain, P12 could inhibit the inflammatory reactions in microglia and neurons, demonstrating its direct neuroprotective effects. This peptide-GNP hybrid nanodevice may represent novel targeted nano-therapeutics to effectively regulate systemic and neuronal inflammation for treating SAE.

## CRediT authorship contribution statement

**Zichen Song:** Writing – review & editing, Writing – original draft, Methodology, Conceptualization. **Hongguang Chen:** Supervision, Project administration, Funding acquisition, Conceptualization. **Wenfei Xu:** Methodology. **Xiaoye Zong:** Methodology. **Xiaoyu Wang:** Methodology. **Yuting Ji:** Methodology. **Jiameng Gong:** Methodology. **Mimi Pang:** Methodology. **Shan-Yu Fung:** Writing – review & editing, Supervision, Funding acquisition. **Hong Yang:** Writing – original draft, Supervision, Project administration, Funding acquisition, Conceptualization. **Yonghao Yu:** Writing – review & editing, Supervision, Project administration, Funding acquisition.

## Ethics approval and consent to participate

All animal experimental protocols were approved by the Institutional Animal Care and Use Committee of Tianjin Medical University General Hospital (No. IRB2023-DW-113).

## Data statement

The datasets used and analyzed during the current study are available from the corresponding author upon reasonable request.

## Funding

This work was supported by the 10.13039/501100001809National Natural Science Foundation of China (grant number 82072150 for YHY; No. 82470080 and No. 82270096 for HY), the Tianjin Health Research Project (grant number TJWJ2023MS001 for HGC), the Key Natural Science Project of 10.13039/501100010882Tianjin Municipal Education Commission (grant number 2023ZD012 for HY) and the 10.13039/501100010229Natural Science Foundation of Tianjin Municipal Science and Technology Commission (21JCYBJC01650 for SYF and 24JCZDJC00650 for HY).

## Declaration of competing interest

The authors declare that they have no known competing financial interests or personal relationships that could have appeared to influence the work reported in this paper.

## Data Availability

Data will be made available on request.
