# Supplementary material for: The hexapeptide functionalized gold nanoparticles protect against sepsis-associated encephalopathy by forming specific protein corona and regulating macrophage activation
Source: Mater Today Bio. 2025 Mar 27;32:101704. doi: 10.1016/j.mtbio.2025.101704 (PMC11997411; doi:10.1016/j.mtbio.2025.101704)
Supplement: Multimedia component 1 [file mmc1.docx]

**Supporting Information for**

**The hexapeptide functionalized gold nanoparticles protect against sepsis-associated encephalopathy by forming specific protein corona and regulating macrophage activation**

Zichen Song^1,#^, Hongguang Chen^1,#^,Wenfei Xu^2^, Xiaoye Zong^2^, Xiaoyu Wang^3^, Yuting Ji^2^, Jiameng Gong^2^, Mimi Pang^2^, Shan-Yu Fung^3^, Hong Yang^2,*^, Yonghao Yu^1,*^

^1^Department of Anesthesia, Tianjin Institute of Anesthesiology, Tianjin Medical University General Hospital, NO. 154 Anshan Road, Tianjin 300052, China

^2^Department of Pharmacology and Tianjin Key Laboratory of Inflammation Biology, The Province and Ministry Co-Sponsored Collaborative Innovation Center for Medical Epigenetics, School of Basic Medical Sciences, Tianjin Medical University, No. 22 Qixiangtai Road, Heping District, Tianjin 300070, China

^3^Department of Immunology and Key Laboratory of Immune Microenvironment and Disease (Ministry of Education), School of Basic Medical Sciences, Tianjin Medical University, No. 22 Qixiangtai Road, Heping District, Tianjin 300070, China

*Correspondence:

Hong Yang

[hongyang@tmu.edu.cn](mailto:hongyang@tmu.edu.cn)

Yonghao Yu

[yyu@tmu.edu.cn](mailto:yyu@tmu.edu.cn)

**List of contents for supporting information**

**1. Supplementary methods**

1.1 LC-MS/MS data analysis

**2. Supplementary figures**

**Figure S1.** The hydrodynamic size distribution of P12.

**Figure S2.** The effects of P12 on neuroinflammation in the brain of mice with CLP-induced sepsis.

**Figure S3.** Total distance moved by the healthy control mice and CLP-induced SAE mice in the Y-maze and NORT.

**Figure S4.** The effects of P12 on the 10-day survival rate of SAE mice.

**Figure S5.** The histological assessment of organ injuries of mice with CLP-induced sepsis with/without P12 treatment.

**Figure S6.** Total distance moved by the healthy control mice and LPS-induced SAE mice in the Y-maze and NORT.

**Figure S7.** The histological assessment of organ injuries of mice with LPS-induced sepsis with/without P12 treatment.

**Figure S8.** The representative images of immune cells in the peritoneal lavage fluid of mice with LPS-induced sepsis with/without the clodronate liposome (Clo-lip) treatment.

**Figure S9.** The representative trajectory images of the mouse moving paths in the behavioral tests on the LPS-induced SAE mice with/without the Clo-lip treatment.

**Figure S10.** The pie charts demonstrate the subcellular localization prediction of differentially enriched proteins in the protein corona of P12.

**Figure S11.** The bubble plots show the GO enrichment of differentially enriched proteins in the protein corona of P12 under the cellular component (CC) categorization.

**Figure S12.** The bubble plots show the GO enrichment of differentially enriched proteins in the protein corona of P12 under the biological process (BP) categorization.

**Figure S13.** The bubble plot shows the KEGG pathway enrichment of differentially enriched proteins in the protein corona on P12.

**Figure S14.** Fluorescence microscopy images of hippocampal cells stained with Iba-1.

**1. Supplementary methods**

**1.1 LC-MS/MS data analysis**

Subcellular localization prediction of differentially expressed proteins (FC > 1.5 for up-regulation, FC < 0.67 for down-regulation, and P < 0.05) was performed using the CELLO (http://cello.life.nctu.edu.tw/) method with a machine learning approach to predict the subcellular localization of the enriched proteins. The GO annotation of enriched proteins was done using Blast2GO. Fisher's exact test was used to compare the distribution of individual GO classifications in the target protein set with the overall protein set to analyze the enrichment of GO annotations.

**2. Supplementary Figures**

**
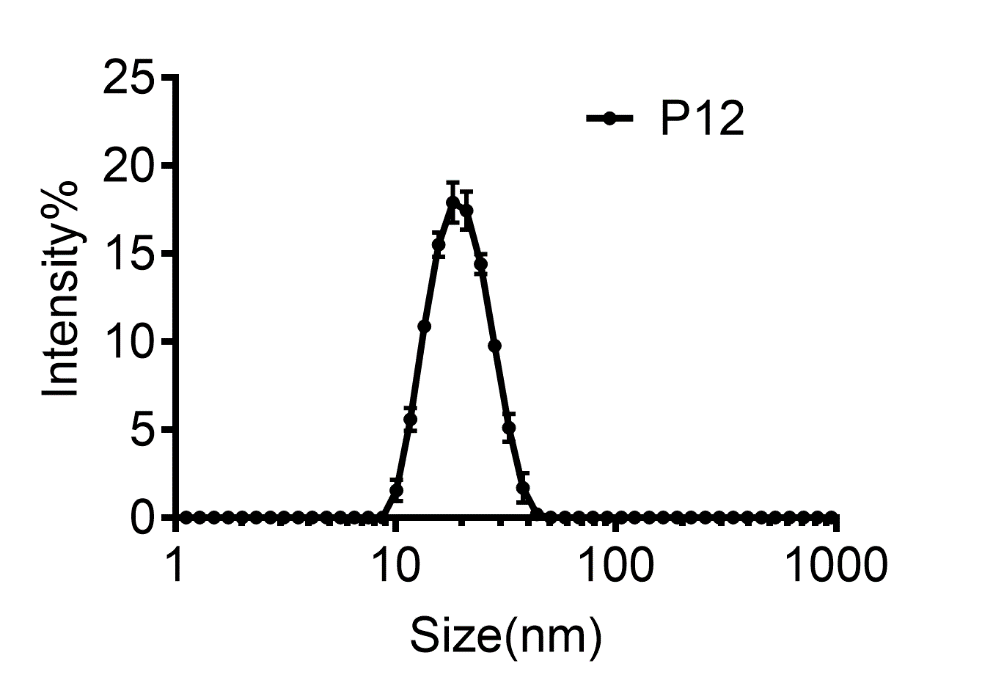
**

**Figure S1.** **The hydrodynamic size distribution of P12.** The intensity-based size distribution of P12 was measured by DLS; N = 3.


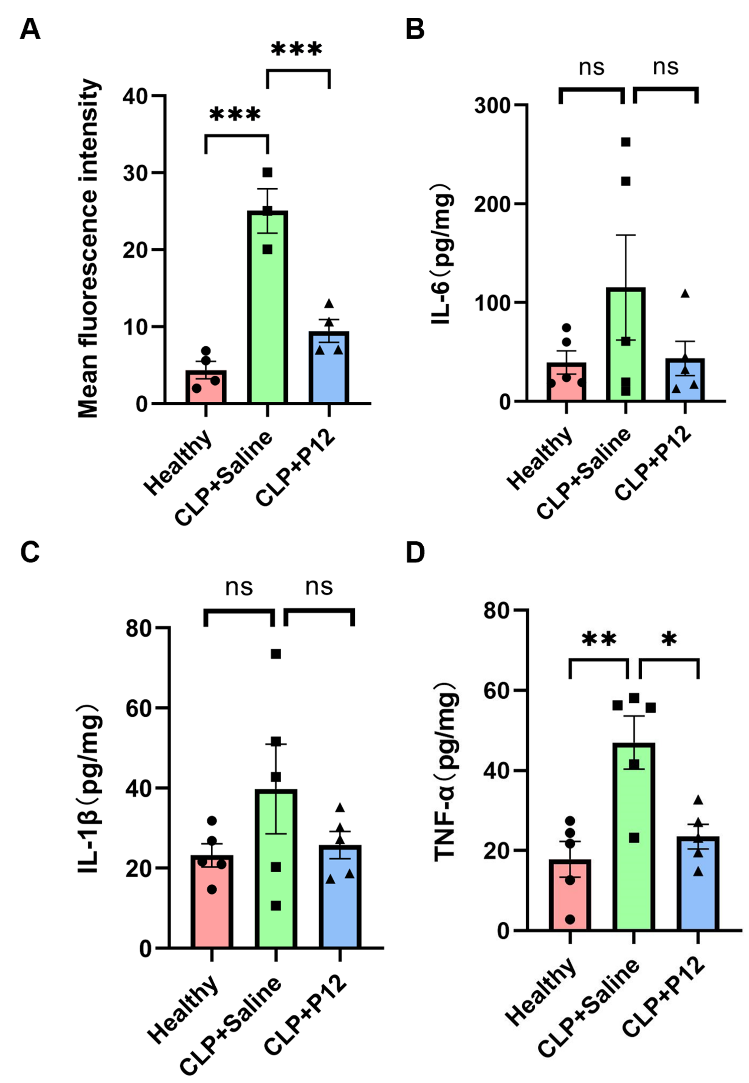


**Figure S2.** **The effects of P12 on neuroinflammation in the brain of mice with CLP-induced sepsis.** (A) The mean fluorescence intensity of the TUNEL staining on the amygdaloid cells was analyzed by the ImageJ software; N = 3-4/group. (B-D) The levels of cytokines IL-6 (B), IL-1β (C), and TNF-α (D) in the surrounding tissues of the hippocampus region at 24 h after CLP induction; N = 5/group. ns: not significant; **P* < 0.05, ***P* < 0.01 and ****P* < 0.001.


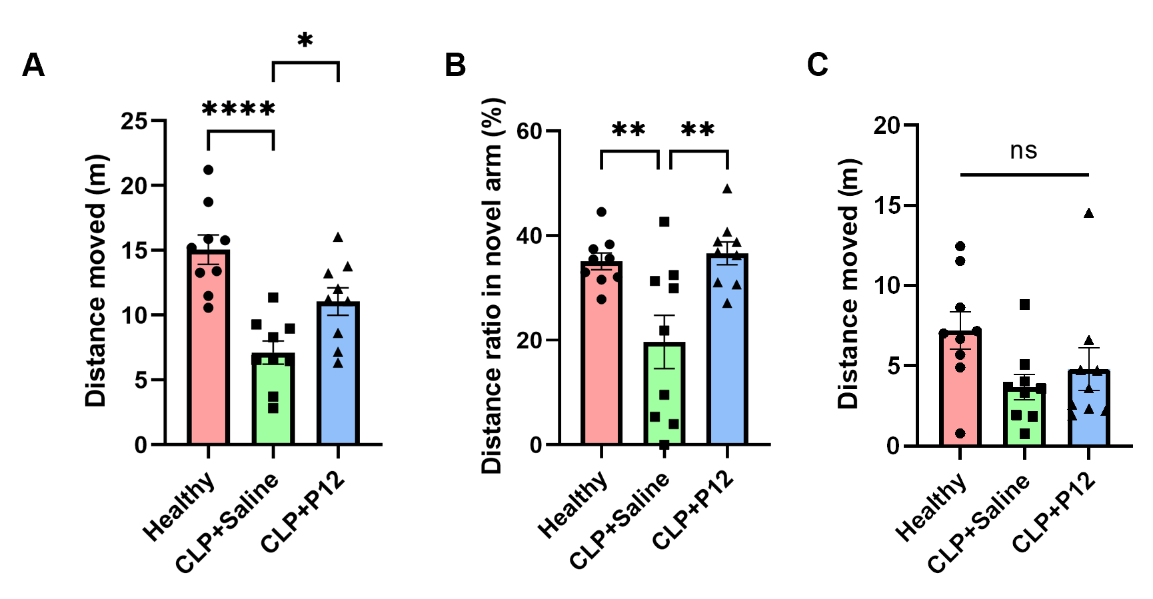


**Figure S3.** **Total distance moved by CLP-induced SAE mice in the Y-maze and NORT.** (A) Quantitative analysis of the distance moved in the Y-maze of CLP-induced septic mice. (B) The ratio of the distance moved in the novel arm to the total distance moved by CLP-induced septic mice. (C) Quantitative analysis of the distance moved in NORT of CLP-induced septic mice. N = 9/group. ns: not significant; **P* < 0.05, ***P* < 0.01, *****P* < 0.0001.


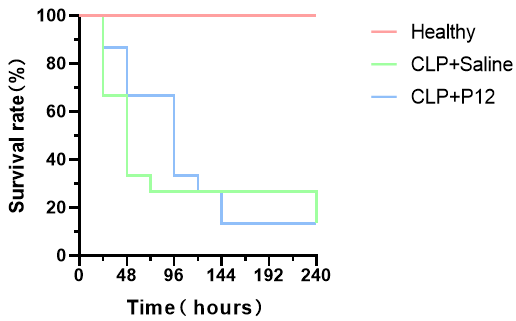


**Figure S4. The effects of P12 on the 10-day survival rate of SAE mice.** The survival of the SAE mice was monitored for 10 days after the CLP procedure with/without P12 treatment; N = 15/group.


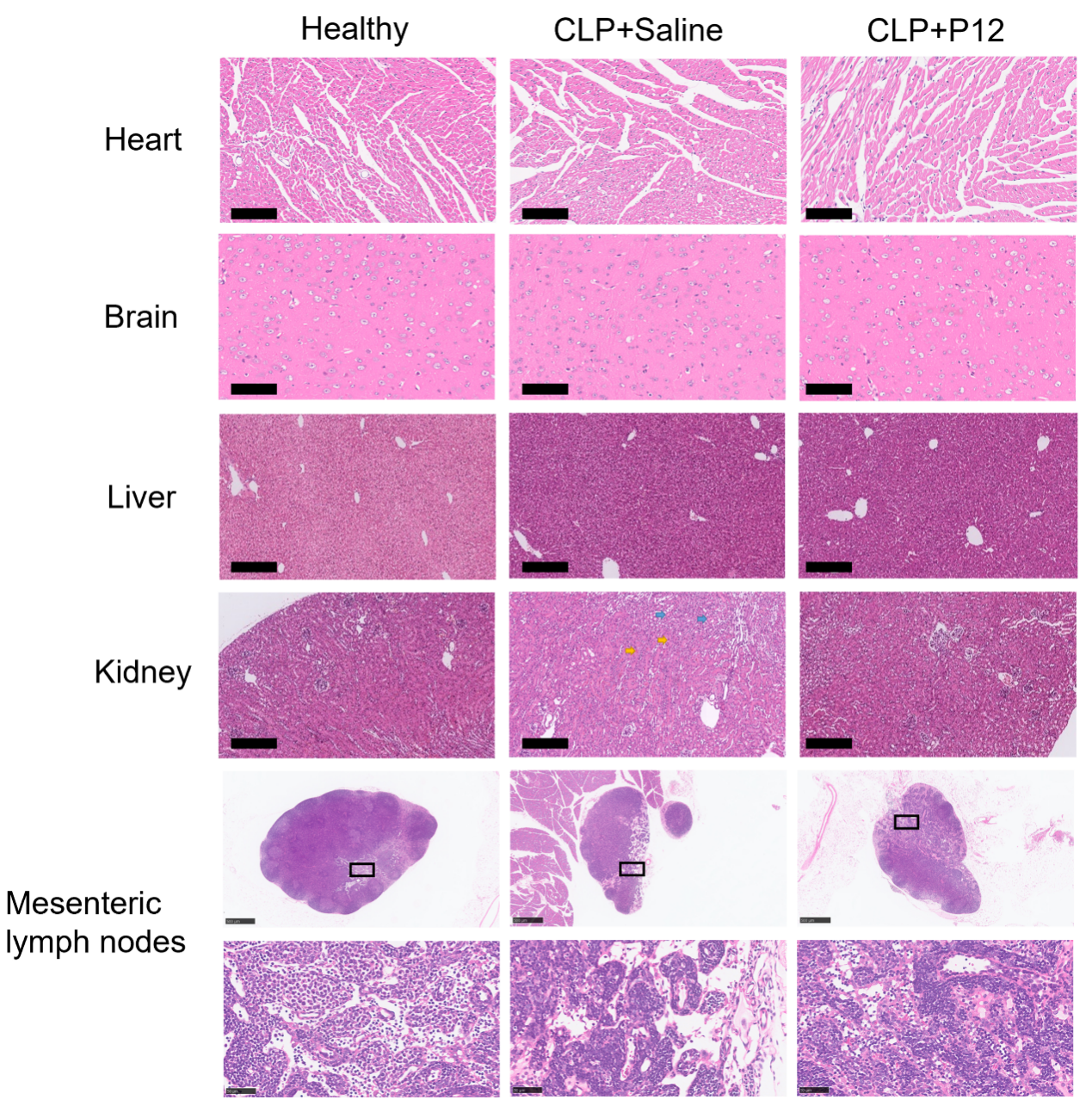


**Figure S5. The histological assessment of organ injuries of mice with CLP-induced sepsis with/without P12 treatment.** After the CLP procedure, the major organs heart, brain, liver, kidneys, and mesenteric lymph nodes were collected and processed for the histological assessment of organ injuries; blue arrows indicated the protein cast and the yellow arrows indicated the acidophilic degeneration in the kidney sections. The scale bar = 200 μm for all the organs except for the mesenteric lymph nodes, where the scale bar = 500 μm (top panel) or 50 μm (bottom panel of the zoom-in images of the black boxes); N = 4-5/group.


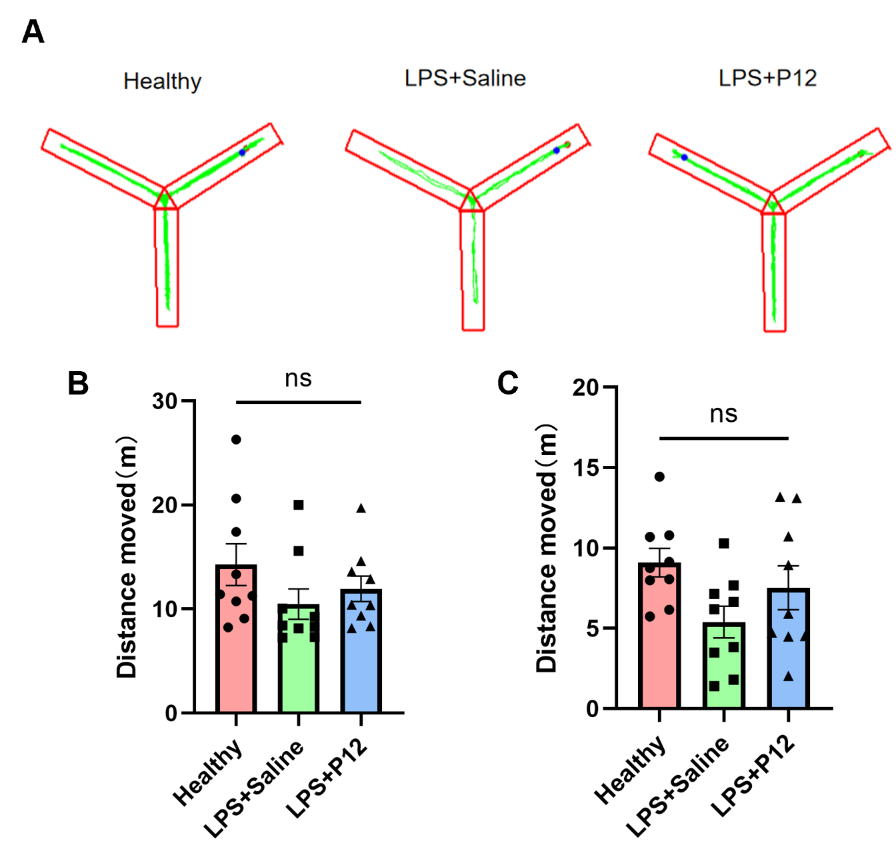


**Figure S6. Total distance moved by LPS-induced SAE mice in the Y-maze and NORT.** (A) The representative records of the walking paths of mice in the Y-maze test. The green lines show the trace of the walking paths of mice in different groups: healthy (left), LPS+saline (middle), and LPS+P12 (right). Quantitative analysis of the distance moved of LPS-induced septic mice in the Y-maze (B) and NORT (C). ns: not significant; N = 9/group.


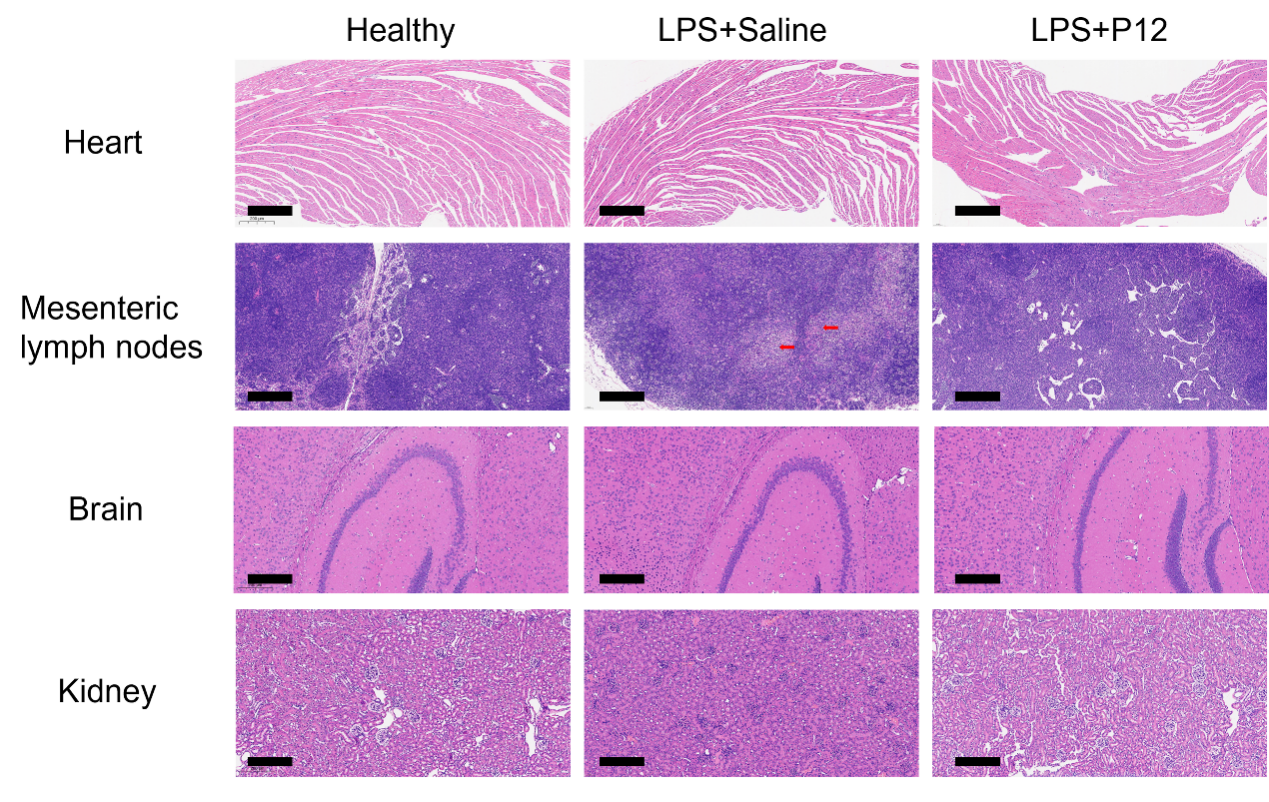


**Figure S7. The histological assessment of organ injuries of mice with LPS-induced sepsis with/without P12 treatment.** After the LPS challenge for 24 h, the major organs heart, brain, kidneys, and mesenteric lymph nodes were collected and processed for the histological assessment of organ injuries; red arrows indicated degenerative necrosis in the mesenteric lymph nodes; scale bar = 200 μm; N = 5/group.


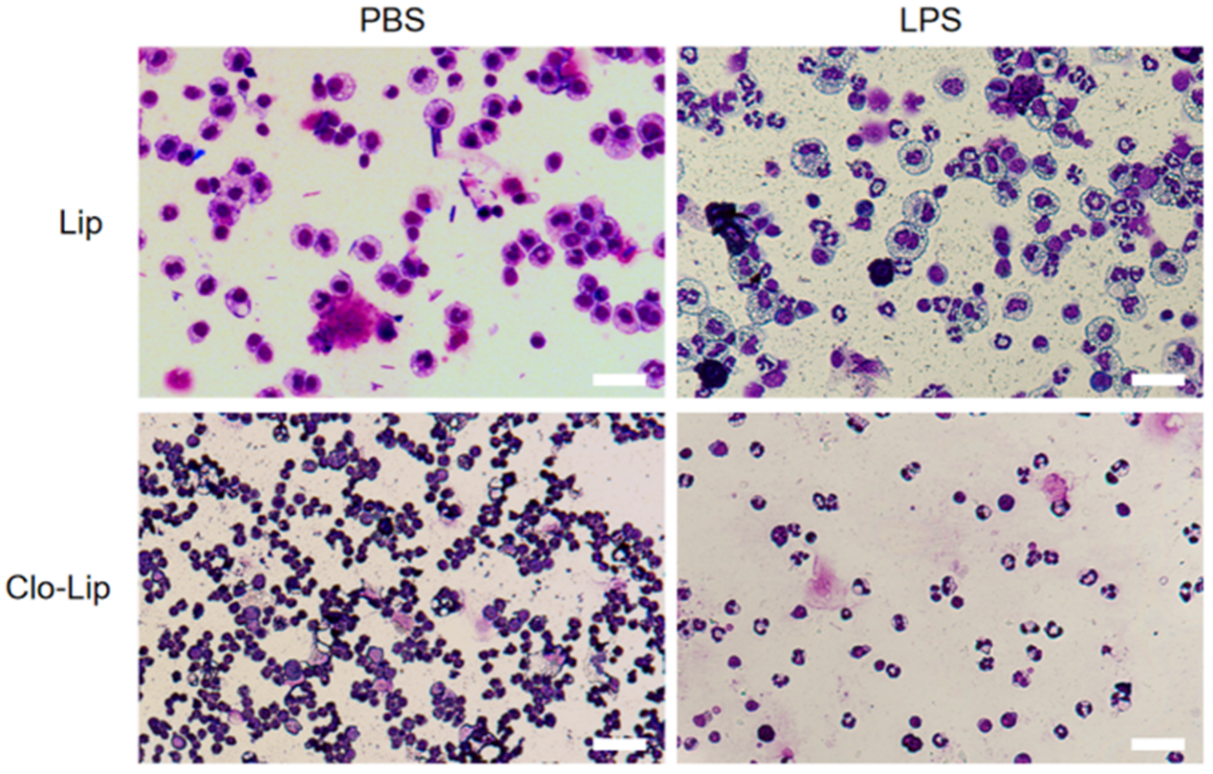


**Figure S8.** **The representative images of immune cells in the peritoneal lavage fluid of mice with LPS-induced sepsis with/without the clodronate liposome (Clo-lip) treatment.** The cell suspensions collected from the peritoneal lavage fluid were cytospined on a glass slide and stained with Liu staining for the differential cell counting on the peritoneal macrophages; scale bar = 30 μm; N = 5/group.


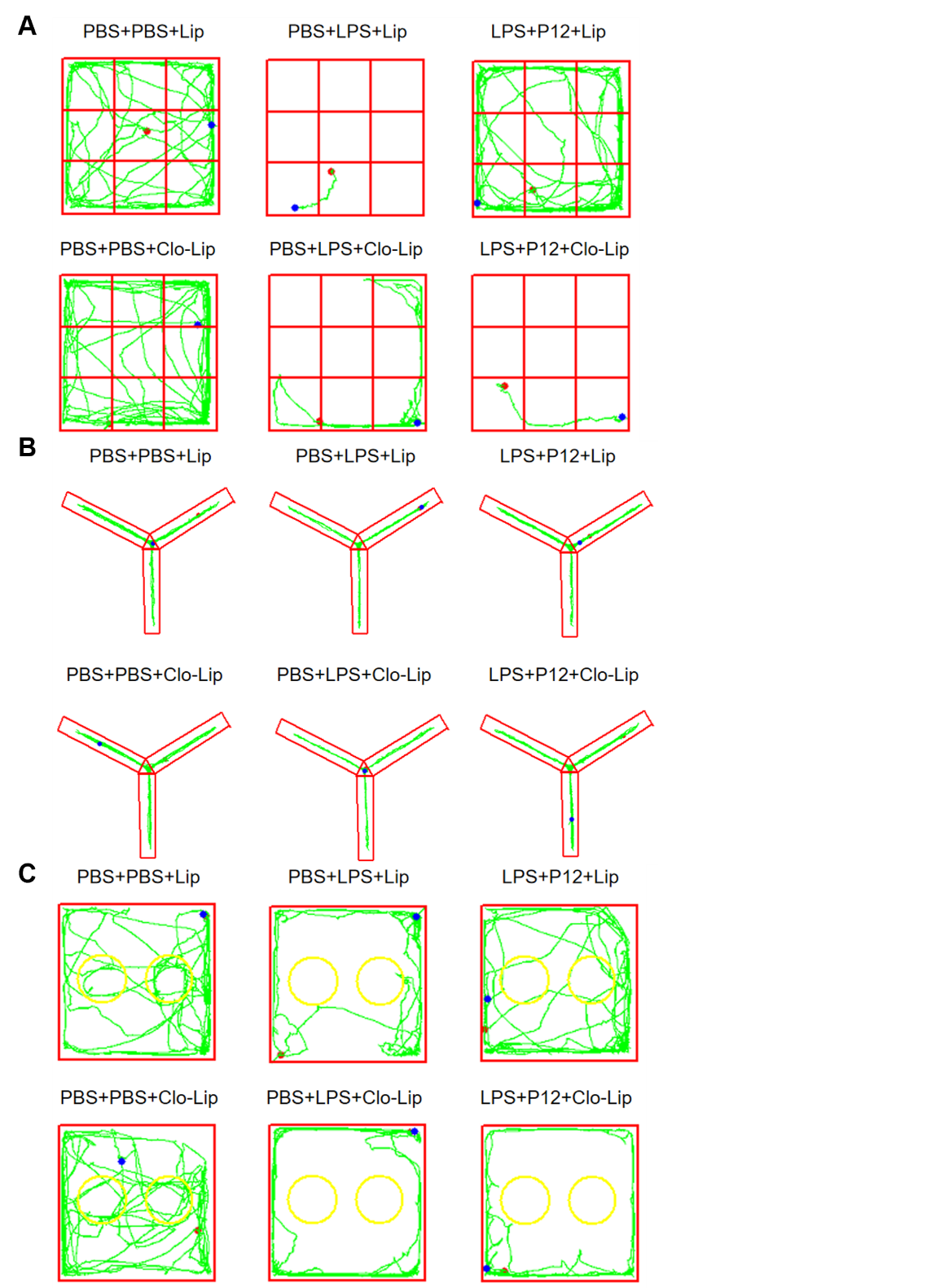


**Figure S9. The representative trajectory images of the mouse moving paths in the behavioral tests on the LPS-induced SAE mice with/without the Clo-lip treatment.** (A-C) The representative images show the mouse moving paths in the OFT (A), the Y-maze test (B), and the NORT (C); N = 5-7/group.


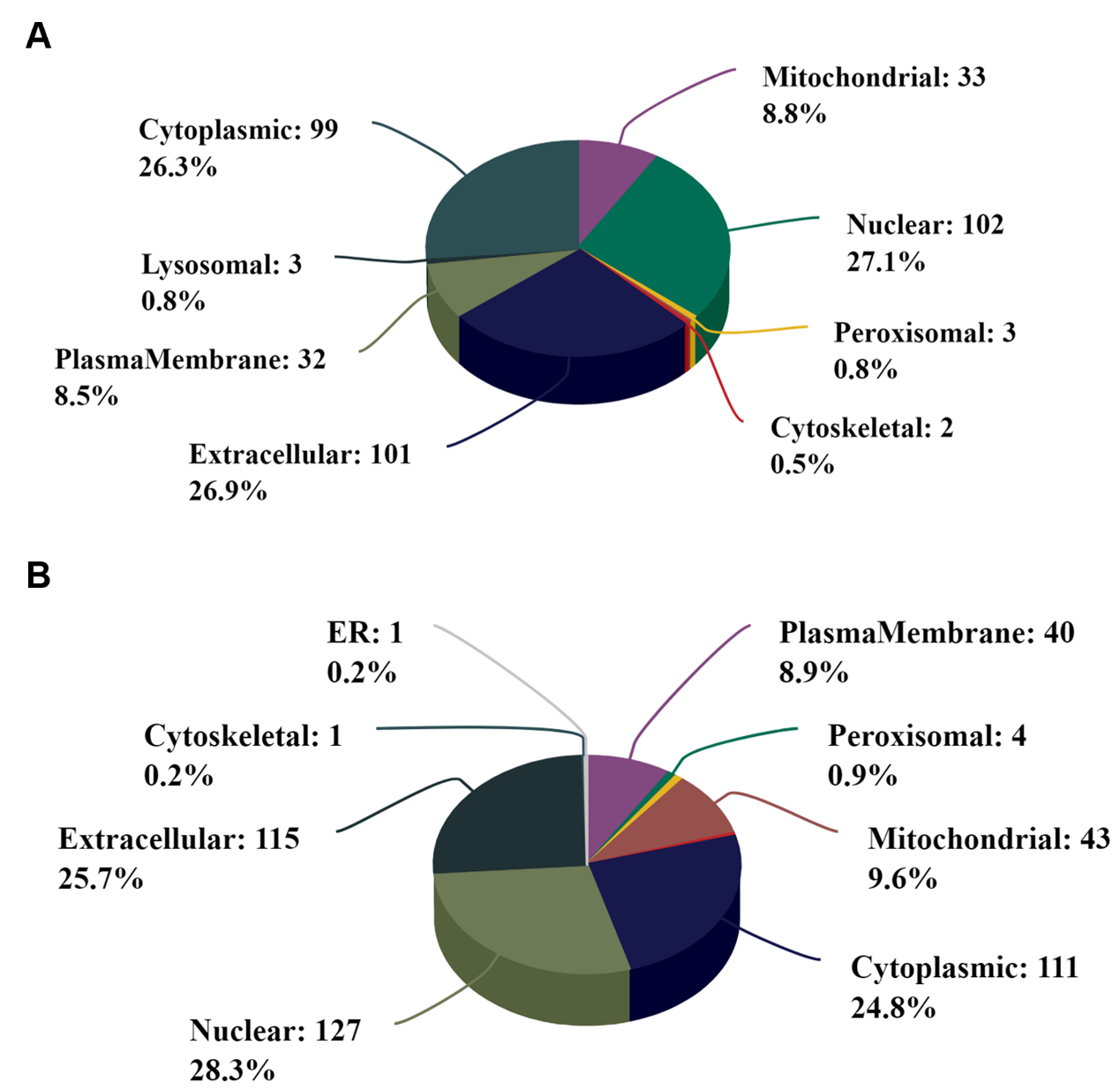


**Figure S10.** **The pie charts demonstrate the subcellular localization prediction of differentially enriched proteins in the protein corona of P12.** The number of proteins and the proportion of their distributions in each organelle were analyzed using the subcellular structure prediction software, CELLO, for the comparison in CLP-P12 vs. CLP-Bare GNP (A), and CLP-P12 vs. Healthy-P12 (B); N = 3/group.


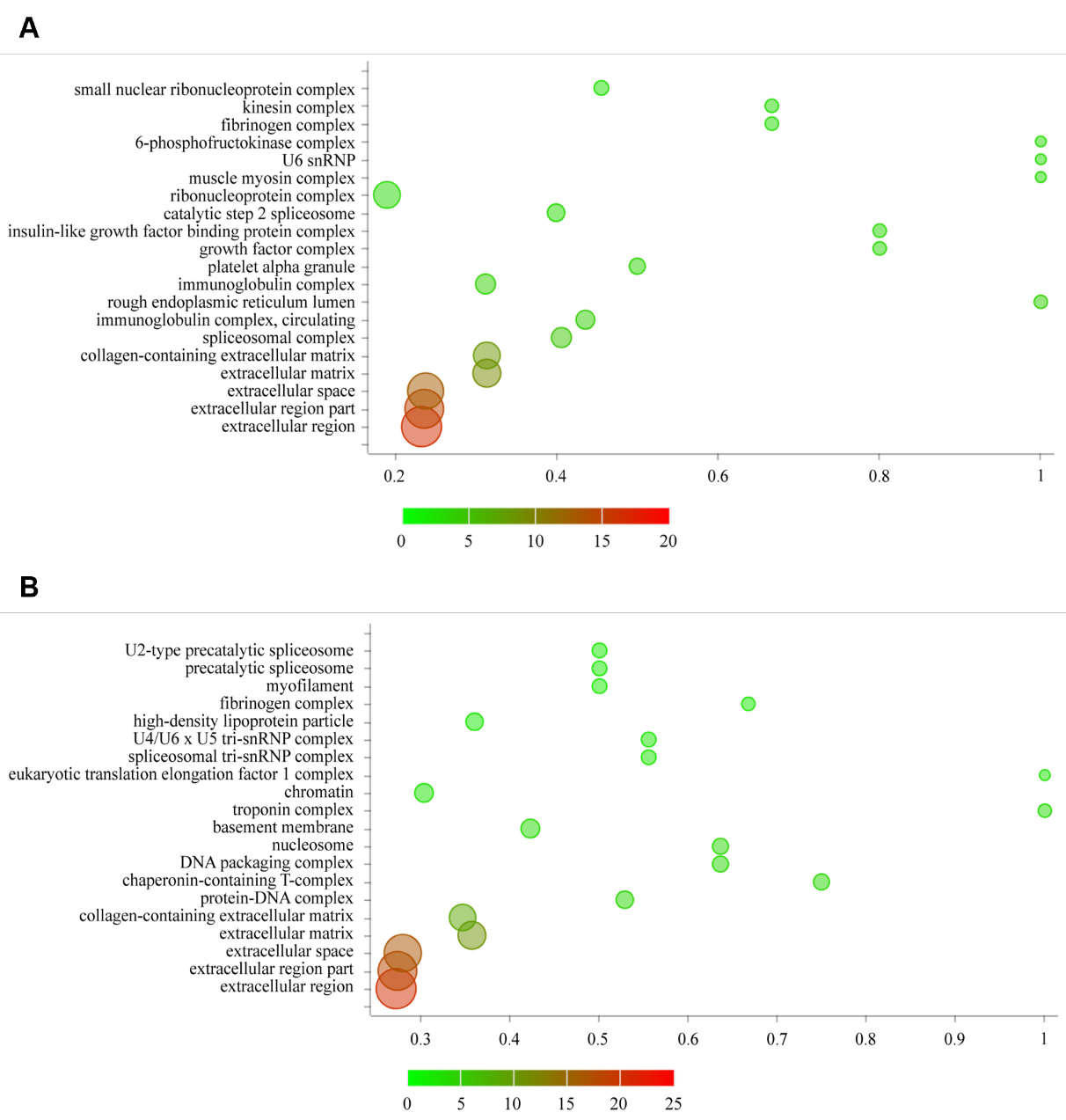


**Figure S11. The bubble plots showing the GO enrichment of differentially enriched proteins in the protein corona of P12 under the cellular component (CC) categorization.** The enrichment of GO entries was analyzed for the comparison in CLP-P12 vs. CLP-Bare GNP (A), and CLP-P12 vs. Healthy-P12 (B); N = 3/group.


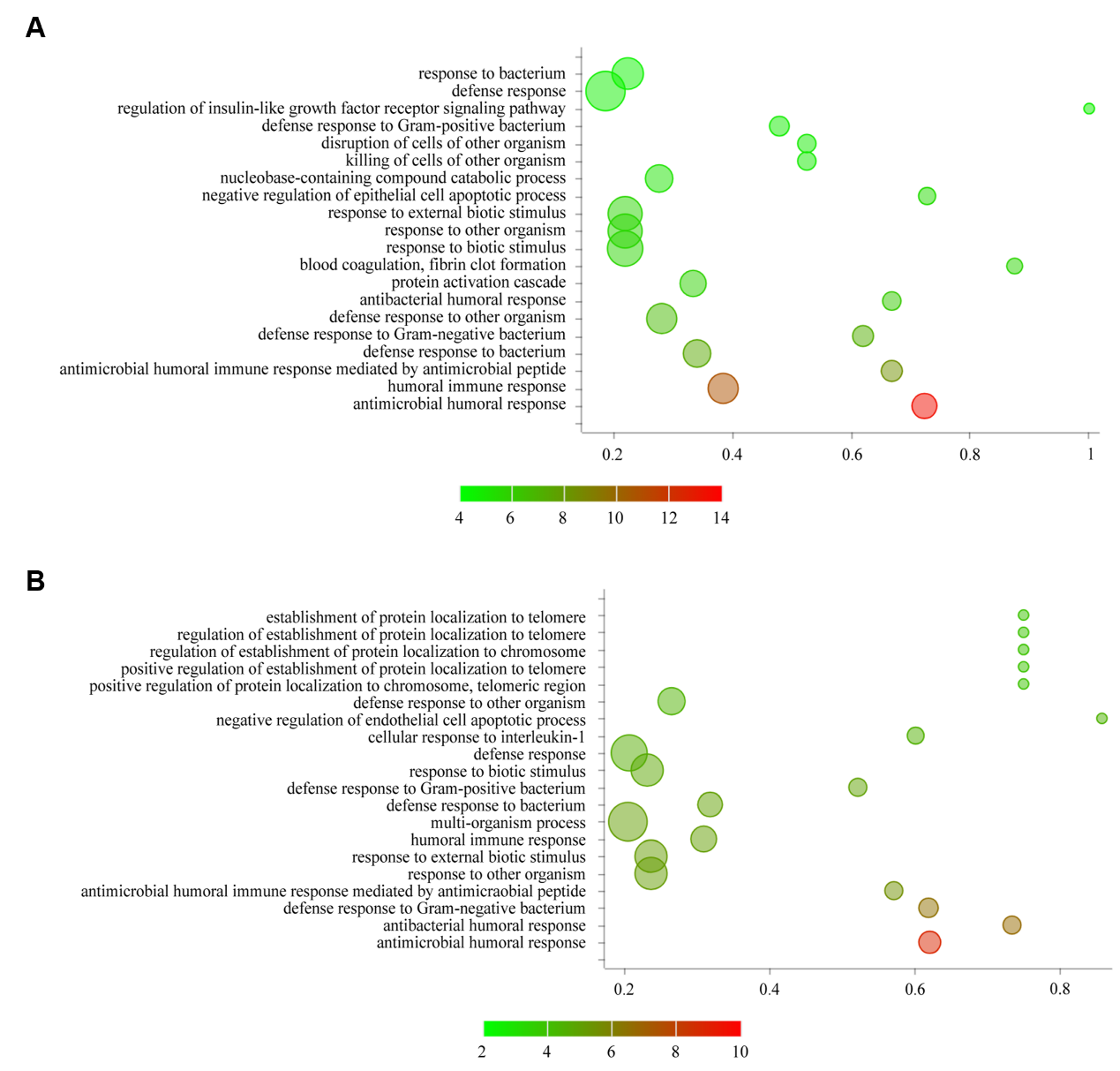


**Figure S12. The bubble plots showing the GO enrichment of differentially enriched proteins in the protein corona of P12 under the biological process (BP) categorization.** The enrichment of GO entries was analyzed for the comparison in CLP-P12 vs. CLP-Bare GNP (A), and CLP-P12 vs. Healthy-P12 (B); N = 3/group.

**
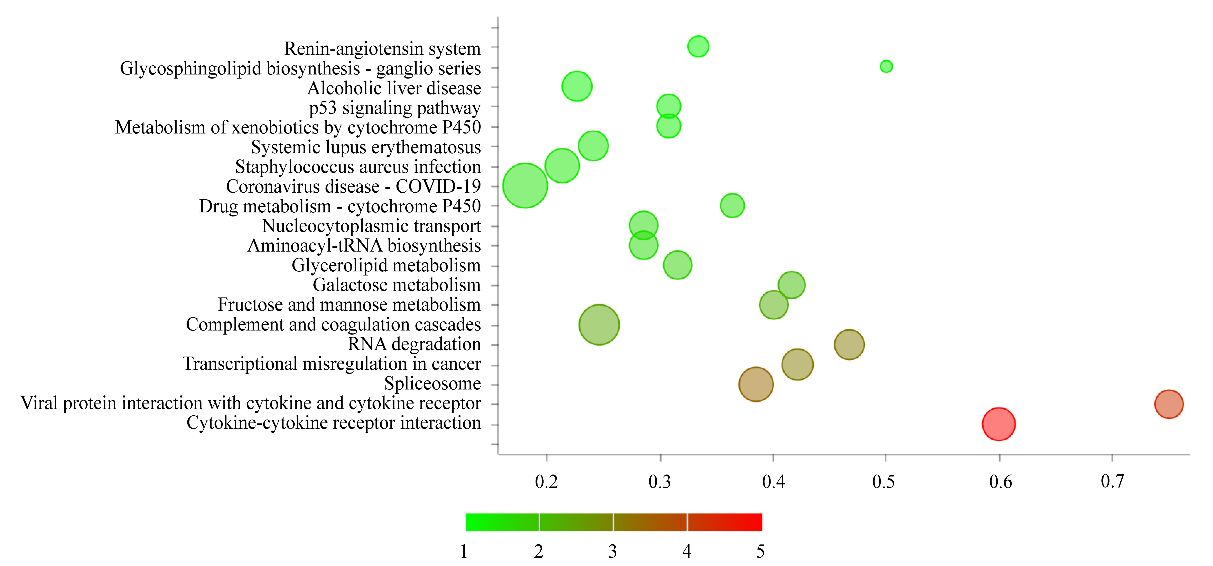
**

**Figure S13. The bubble plot shows the KEGG pathway enrichment of differentially enriched proteins in the protein corona on P12.** The analysis was done for the comparison of CLP-P12 vs. CLP-Bare GNP; N = 3/group.


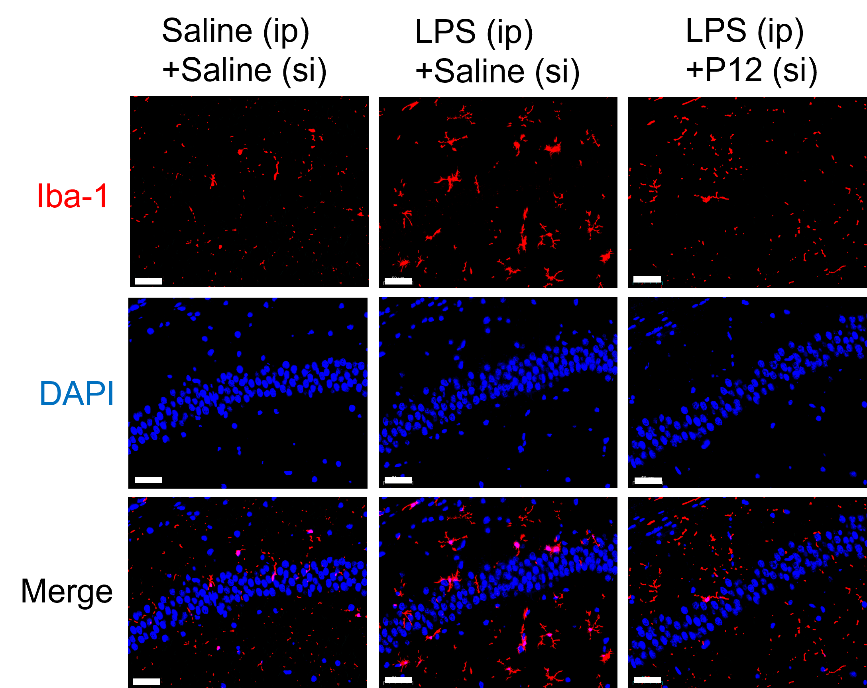


**Figure S14.** Fluorescence microscopic images of hippocampal cells in the brain sections stained with anti-Iba-1 antibody (red) 24 h post-LPS challenge; the nuclei were stained with DAPI (blue); scale bar = 50 μm. N = 4-5/group.
